# Visualization of stepwise derepression of TFIIH in global genome nucleotide excision repair

**DOI:** 10.1126/sciadv.aeb3506

**Published:** 2026-07-23

**Authors:** Natàlia de Martín Garrido, Callum A. F. Haste, Junjie Feng, Nora B. Cronin, Basil J. Greber

**Affiliations:** ^1^ Division of Structural Biology, The Institute of Cancer Research, 237 Fulham Road, London SW3 6JB, UK.; ^2^ London Consortium for High Resolution Cryo-EM, The Francis Crick Institute, London NW1 1AT, UK.

## Abstract

Nucleotide excision repair (NER) is a crucial DNA repair pathway that is orchestrated by transcription factor IIH (TFIIH) in eukaryotic cells. TFIIH is a multifunctional complex that contains two DNA helicase/DNA translocase subunits and a kinase module, different subsets of which act in NER, transcription initiation, and cell cycle control. To ensure fidelity despite multifunctionality, the DNA helicase activity of TFIIH is autoinhibited in its free form or when the factor engages in transcription initiation. While the release of the kinase module has been identified as a key step in TFIIH activation, the molecular mechanisms controlling this step and concomitant structural changes in TFIIH are incompletely understood. Here, we determine high-resolution structures of three NER intermediates that visualize how TFIIH arrives at sites of DNA damage in an autoinhibited state and how autoinhibition is released via previously undescribed intermediates. These findings contribute to a mechanistic understanding of human DNA repair.

## Teaser

Cryogenic electron microscopy captures nucleotide excision repair complexes assembling on damaged DNA templates.

## INTRODUCTION

Nucleotide excision repair (NER) is responsible for removal of bulky base lesions originating from the exposure of cellular DNA to ultraviolet (UV) radiation or reactive chemicals, including cis-platin and related cancer therapeutics [reviewed in (*1*, *2*)]. NER deficiencies are involved in human disorders (*3*, *4*), including trichothiodystrophy, Cockayne syndrome, and Xeroderma pigmentosum (XP). The latter is the namesake for the XP proteins (XPA to XPG), which are critical for NER. In addition, the pathway has a propensity to interfere with cancer therapy (*5*, *6*), making it a possible target for sensitizing cancer cells to DNA-damaging agents. Therefore, NER has been intensively studied by biochemical, cell biological, and structural methods.

Two subpathways known as global genome NER (GG-NER) and transcription-coupled NER use dedicated protein complexes or stalled RNA polymerase II (Pol II) as DNA damage sensors, respectively [reviewed in (*7*, *8*)]. In GG-NER, DNA damage is recognized by the UV-damaged DNA binding protein (UV-DDB) complex (DDB2 and DDB1, which additionally recruit Cullin 4A and RING-box protein 1) (*9*–*12*) and the XPC complex (XPCc; XPC, human RAD23 homolog B or hRAD23B, and Centrin 2 or CETN2) (*13*–*16*), which initiate a complex cascade of molecular processes at the DNA lesion. Critically, XPCc recruits transcription factor IIH (holo-TFIIH) to the DNA lesion by formation of direct protein-protein interactions (*17*, *18*). Holo-TFIIH is a 10-subunit transcription and DNA repair factor that is subdivided into a 7-subunit core complex (XPD, XPB, p62, p52, p44, p34, and p8) and a 3-subunit cyclin-dependent kinase (CDK)–activating kinase (CAK) subcomplex (CDK7, cyclin H, and MAT1) (*19*). XPB is a superfamily 2 (SF2) double-stranded DNA (dsDNA) translocase required for both transcription initiation and NER (*20*), while the enzymatic activity of the SF2 DNA helicase XPD is restricted to NER (*21*). It is thought that XPD is the active TFIIH helicase in NER, while XPB primarily contributes to DNA repair via its adenosine triphosphatase (ATPase) activity (*22*). In holo-TFIIH, XPD activity is repressed by the CAK subunit MAT1 (*23*–*27*), and its DNA and adenosine 5′-triphosphate (ATP)–binding sites are blocked by the TFIIH core subunit p62 (*28*, *29*). Derepression of XPD by the release of CAK has been shown to require both ATP and the NER factor XPA, which joins the lesion site after TFIIH (*30*). XPA binds to the single-stranded DNA (ssDNA)–dsDNA junction on the 5′ side of the lesion (*25*, *31*) and plays multiple roles during preincision complex assembly: It is involved in lesion verification (*27*), and it interacts with replication protein A (RPA) (*32*) and the DNA endonuclease XPF–excision repair cross-complementing 1 (XPF-ERCC1) (*13*, *33*, *34*). Following XPD derepression, the XPD and XPB DNA helicase and DNA translocase subunits of TFIIH cooperate to unwind the DNA around the site of the lesion (*22*, *27*). The helicase activity of XPD is stimulated by the DNA endonuclease XPG (*25*), which binds to XPD itself during assembly of the preincision complex (*25*, *35*). Licensing of dual incision after DNA unwinding requires additional verification of the presence of a bona fide NER lesion (*36*) and is thought to depend on stalling of DNA unwinding when a bulky DNA lesion is encountered by XPD (*27*, *37*, *38*). The incisions on the 3′ and 5′ sides of the lesion are performed by the DNA endonucleases XPG and XPF-ERCC1, respectively, with XPF-ERCC1 cutting first (*39*). RPA, covering and thereby protecting the undamaged strand of the DNA, additionally aids in placement or activation of XPF-ERCC1 (*40*, *41*) and XPG (*42*, *43*). XPG and RPA are also involved in recruitment of proliferating cell nuclear antigen (PCNA) and replication factor C (RFC) (*44*), thereby enabling gap-filling DNA synthesis, which involves DNA polymerases ε or δ/κ (*45*).

NER is thus a dynamic and intricate multistep process in which multiple DNA repair factors are recruited and subsequently activated in a tightly controlled fashion. This ensures fidelity of the repair process and prevents unnecessary, harmful DNA incisions. There is a critical requirement for high-resolution structural information to obtain a mechanistic understanding of the molecular transitions involved in assembly of the preincision complex. Uncovering the mechanisms by which the dynamic interplay of repair factors at the DNA lesion results in controlled activation of the required enzymatic activities is of particular interest. Several multifactor GG-NER intermediates and DNA-bound subassemblies have been characterized by x-ray crystallography (*12*, *16*, *46*) and cryo–electron microscopy (cryo-EM) (*25*, *47*–*52*), and purely computational studies have provided information about the parts of the process that have been previously intractable by experimental structural methods (*35*). However, characterization of early steps of the NER process after TFIIH recruitment has remained incomplete because of the absence of the CAK subcomplex from TFIIH in several studies, and the ability to resolve dynamic transitions might have been affected by the presence of cross-linking agents during sample preparation for cryo-EM.

Here, we implement a structure determination strategy based on streptavidin affinity grids (*53*, *54*), thereby obviating the need for cross-linking and facilitating the structural characterization of dynamic intermediates. We use this system to reconstitute the early stages of human GG-NER, including holo-TFIIH in the presence of its CAK subcomplex. Our structures reveal initial recruitment of an autoinhibited TFIIH complex to DNA lesions, followed by stepwise derepression of TFIIH activity by the protein XPA and by dynamic conformational rearrangements within TFIIH. In doing so, we visualize three NER intermediates, providing evidence that previous models of the repair process did not capture the full complexity of the molecular processes involved, and rationalize prior data suggesting a requirement for ATP in the release of the TFIIH-inhibitory CAK.

## RESULTS

### Structure determination

**Fig. 1.**
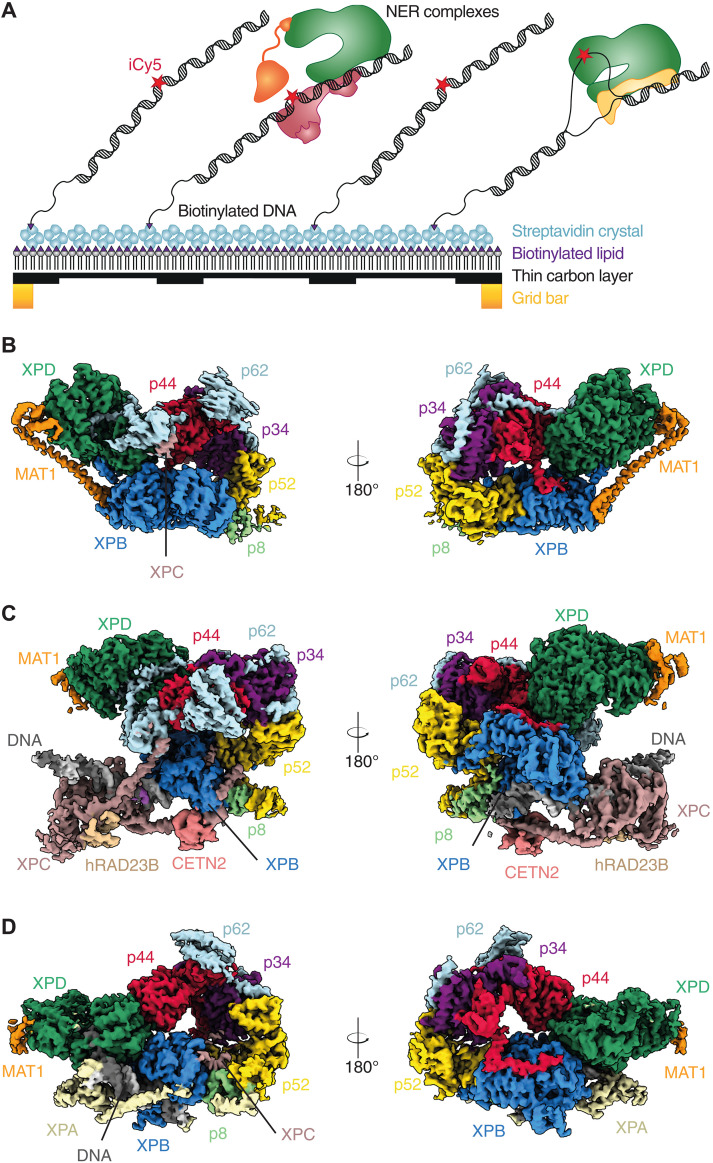
Cryo-EM reconstructions of three GG-NER complexes from streptavidin affinity grids. (**A**) Principle of the capture of NER complexes on streptavidin affinity grids using biotinylated DNA. (**B** to **D**) Two views of the 3.3-Å resolution holo-TFIIH-XPC initial encounter complex (IEC) reconstruction (B), the composite map of holo-TCD complex (C), and the 3.6-Å resolution holo-TCDA complex reconstruction (D). Maps are color coded and labeled according to their constituent subunits.

Because of the fragility of many large molecular assemblies, their dissociation upon contact with the air-water interface during cryo-EM grid preparation poses challenges during structure determination [e.g., (*55*)]. To avoid the use of cross-linking agents, which have often been used to counteract these destructive effects and stabilize NER assemblies for structure determination (*25*, *47*), we adapted the streptavidin affinity grid system for the study of this process (*53*, *54*). We used biotinylated DNA harboring an internal cyanin-5 (iCy5) modification, as used in previous studies of NER complexes (*47*), to tether molecular complexes assembling around this DNA lesion to the cryo-EM grids (Fig. 1A and fig. S1A). Unbound complexes were washed off before vitrification after a brief incubation period (see Materials and Methods). To study recruitment and activation of TFIIH during GG-NER, we recombinantly expressed and purified the 10-subunit holo-TFIIH complex including the CAK subcomplex, the trimeric XPCc, and XPA (figs. S1, B to E, and S2A). Binding of purified components to the biotinylated damaged DNA was confirmed by electrophoretic mobility shift assay (fig. S2B). We initially bound a specimen containing holo-TFIIH, XPCc, and DNA to streptavidin affinity grids (fig. S3A). Having verified the presence of high-quality two-dimensional (2D) classes after streptavidin lattice subtraction (fig. S3, A and B), we were able to reconstruct the 3D structure of two different complexes—a complex resembling free holo-TFIIH and a holo-TFIIH-XPCc-DNA (holo-TCD) complex—tethered to the streptavidin surface (fig. S4). To further investigate the activation of TFIIH in early GG-NER, we added XPA to the specimen containing holo-TFIIH, XPCc, and DNA. We collected approximately 80,000 cryo–electron micrographs from these holo-TFIIH-XPC-XPA-DNA grids (fig. S5, A and B) and used cryoSPARC (*56*) and RELION (*57*, *58*) for high-resolution structure determination (figs. S6 to S10). We were again able to reconstruct a complex resembling free TFIIH and the holo-TCD complex (Fig. 1, B and C, figs. S7 to S9, and tables S1 to S3), which we had also identified on the holo-TFIIH-XPC-DNA grids. In addition, we were able to resolve a holo-TFIIH-XPCc-DNA-XPA (holo-TCDA) complex (Fig. 1D, figs. S6 and S10, and tables S1 to S3).

### Structure of the holo-TFIIH-XPCc initial encounter complex

**Fig. 2.**
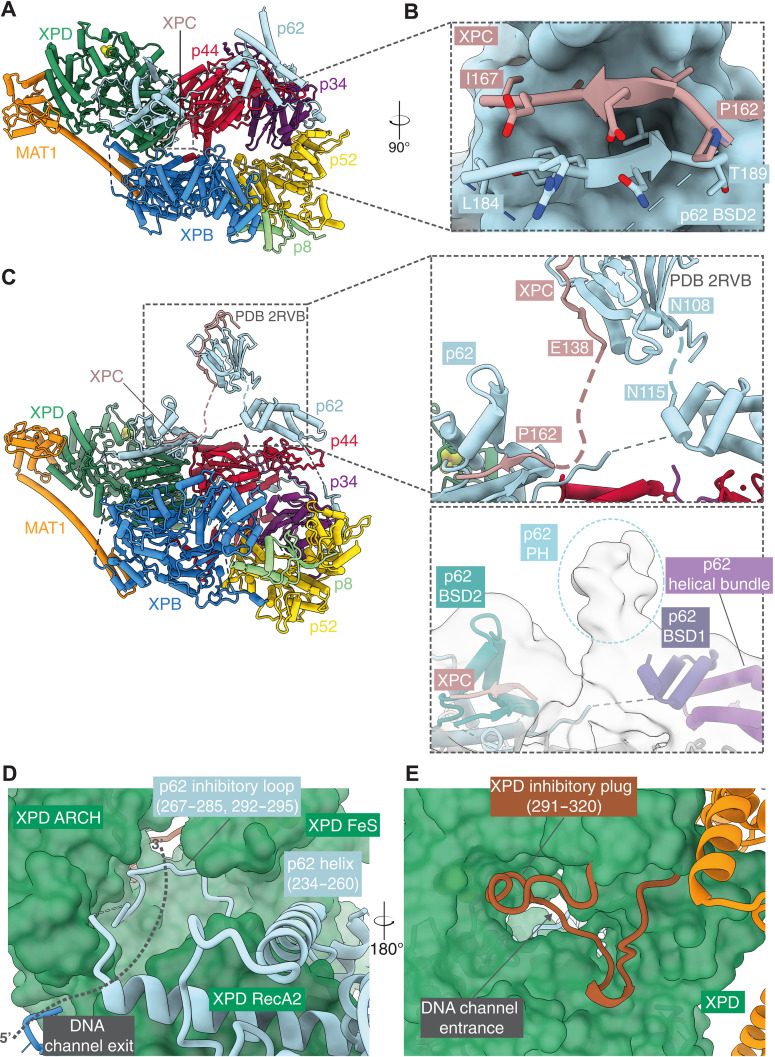
XPCc and holo-TFIIH form an IEC. (**A**) Cartoon representation of the atomic model of the holo-TFIIH-XPC IEC, colored by subunit, with metal ions and the FeS cluster depicted as spheres. (**B**) Close-up view of the interaction between XPC residues 162 to 167 and the BSD2 domain of p62. (**C**) The XPC acidic string bound to the Pleckstrin homology (PH) domain of p62 [Protein Data Bank (PDB) 2RVB (*17*); not built in our model due to low local map quality], positioned in place of density seen adjacent to the p62 BSD1 and BSD2 domains. The density is filtered to 8-Å resolution for visualization. (**D** and **E**) Views of the XPD DNA channel, viewed from the exit (D) and entry (E) sites, with XPD shown as a green surface and inhibitory regions shown as cartoons (p62 loop: light blue; XPD plug: brown).

Only molecular assemblies interacting with DNA were expected to remain tethered to our cryo-EM grids due to washing steps during grid preparation. However, as outlined above, one of the three high-resolution structures obtained from our streptavidin affinity grids showed very strong resemblance to free TFIIH (Fig. 1B and figs. S7 and S11A). Careful analysis of the cryo-EM map at 3.3-Å overall resolution (fig. S8) revealed density for a short protein segment bound to the second BTF2-like transcription factors, synapse-associated proteins, and DOS2-like proteins (BSD2) domain of the TFIIH subunit p62 (fig. S11B). AlphaFold 3 predictions (fig. S11, C to F, and data S1) indicate that this density is occupied by an XPC segment (residues 162 to 167; fig. S11, C and D). The validity of this AlphaFold 3 prediction is supported by the predicted aligned error (PAE) plot (fig. S11C), by the agreement between prediction and cryo-EM map (fig. S11D), by the fact that the presence of this XPC segment in this location is consistently predicted (fig. S11E), and by the visualization of neighboring segments of XPC in matching locations in the holo-TCD complex (fig. S11D and see below). Together, these observations suggest that this structure represents an initial encounter complex (IEC) in which TFIIH interacts with DNA-bound XPC but is not yet binding to DNA itself (Fig. 2, A and B).

The acidic string of XPC (residues 109 to 156) has been shown to interact with the Pleckstrin homology (PH) domain of p62 (residues 1 to 108) previously (*17*). The additional interaction between XPC and the TFIIH BSD2 domain observed in our cryo-EM reconstruction is mostly governed by hydrogen bonds, with XPC residues 162 to 167 forming an antiparallel β sheet with the BSD2 domain of p62 (Fig. 2B). After low-pass filtering our cryo-EM reconstruction of the holo-TFIIH-XPC IEC to 8 Å, we could observe weak density for the PH domain of p62 in the vicinity of the p62 BSD2 domain (Fig. 2C). The interaction of XPC residues 162 to 167 with the p62 BSD2 domain is thus sterically compatible with the simultaneous formation of the well-documented interaction of the XPC acidic string with the p62 PH domain (Fig. 2C). Given that the XPC acidic string is able to stably bind to p62 on its own, the interaction formed by XPC residues 162 to 167 might be of lower affinity and primarily aid in positioning of TFIIH relative to XPC in the holo-TCD complex (see below) rather than serving in TFIIH recruitment as such.

Except for the visualized XPC segment, our structure of this holo-TFIIH-XPC IEC at 3.3-Å resolution is very similar to our previous 3.7-Å resolution structure of free TFIIH determined using a Volta phase plate (*28*), confirming that our recombinant TFIIH complex is structurally and compositionally equivalent to complexes purified natively from HeLa cells (fig. S11A). All seven TFIIH core subunits are visualized, along with N-terminal portions of the CAK subunit MAT1, which connects the XPD ARCH domain to the XPB DNA damage recognition domain (DRD) (Figs. 1B and 2A and fig. S12A). Our previous structure of free TFIIH revealed segments of p62 occluding the active site and DNA binding surfaces of XPD, thereby acting in concert with MAT1 to inhibit XPD activity in the context of transcription initiation (*28*). However, due to limited local resolution, it was not possible to assign the sequence to some of these segments. On the basis of improved side-chain density in our cryo-EM map of the holo-TFIIH-XPC IEC, we were able to assign the sequence register of several of these segments (fig. S12, B to D), revealing the detailed molecular contacts that mediate XPD inhibition within free holo-TFIIH. These regions include the negatively charged inhibitory XPD anchor loop of p62 (residues 267 to 285 and 292 to 295; Fig. 2D and fig. S12, C to E) that is inserted in the DNA binding cavity of XPD and an α helix formed by p62 residues 234 to 260 (fig. S12C). This α helix binds to the surface of the XPD RecA2 domain and connects the p62 BSD2 domain with the inhibitory loop of p62 blocking the DNA binding cavity of XPD (Fig. 2D). Furthermore, the DNA binding channel of XPD is blocked by an XPD autoinhibitory plug emanating from within its own ARCH domain (Fig. 2E). Previous structures of free TFIIH exhibited weak density for this plug, which is better resolved in our cryo-EM map of holo-TFIIH-XPC IEC, allowing us to build a more detailed model (fig. S12B).

### Visualization of autoinhibited TFIIH after recruitment to an NER lesion

**Fig. 3.**
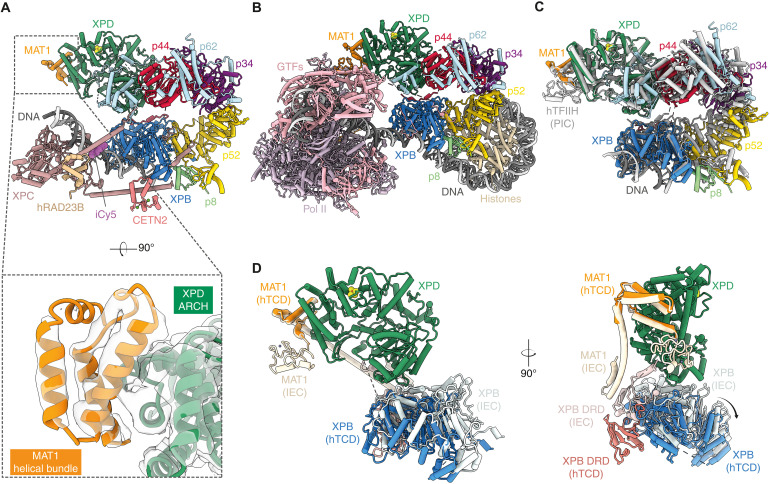
Holo-TFIIH is recruited at the lesion site in an autoinhibited state. (**A**) Cartoon representation of the atomic model of the holo-TCD complex, colored by subunit and with ligands and the iCy5 moiety depicted as spheres. Inset: Close-up view of density for the MAT1 helical bundle bound to the XPD ARCH domain. The map represented was obtained by multibody refinement (body 1) and was low-pass filtered to 4 Å for visualization. (**B**) Full atomic model of the Pol II preinitiation complex (Pol II–PIC) showing engagement of DNA by XPB [PDB 8BVW (*63*)]. GTFs, General transcription factors. (**C**) Superposition of the TFIIH subunits of holo-TCD (in colors) and the Pol II–PIC (in light gray). (**D**) Superposition of the holo-TCD complex (subunits labeled hTCD) and the holo-TFIIH-XPC IEC (subunits labeled IEC) showing the XPB and XPD helicases and MAT1 to visualize the structural changes in XPB upon DNA engagement by XPB. The highly mobile XPB DRD is highlighted in pink hues (light pink: holo-TFIIH-XPC IEC; dark pink: holo-TCD complex).

The structure of holo-TFIIH in complex with lesion-bound XPCc (holo-TCD complex), determined from the same cryo-EM grids as the holo-TFIIH-XPC IEC (figs. S7 and S9), is highly dynamic. Accordingly, determination of this structure at a resolution that allowed reliable model building required the use of multibody refinement (fig. S13, A and B, and table S2) (*59*). Multibody refinement using three masks centered on XPD, XPB, and XPCc (fig. S13A) followed by principal component analysis shows continuous rotations of the XPD and XPB regions of the complex relative to each other, with the p52-p34 region serving as a hinge point (fig. S13C). At the same time, the XPCc and DNA downstream of the lesion (i.e., distal to TFIIH) are seen to swing away from the core of the complex, hinging around the flexible lesion area, overall indicating a highly dynamic structure. Despite the dynamic nature of this complex, multibody refinement allowed us to obtain interpretable maps, substantially improved over the initial consensus refinement (figs. S9, D and G, and S13, D to H). We were able to build a molecular model comprising the seven TFIIH core subunits, the N-terminal portion of MAT1, and portions of XPC, CETN2, and hRAD23B along with 35 nucleotides of DNA (Fig. 3A and fig. S14A). The DNA stretches from XPB, which is bound to dsDNA, to XPC, which recognizes the presence of the lesion. The DNA beyond the lesion site is mobile and shows modest map quality even in the multibody refined maps. The position of the XPC-DNA portion in our reconstruction deviates from that observed in a previous core-TFIIH-XPC-DNA (core-TCD structure) (*47*), in line with the conformational dynamics observed in our dataset (fig. S14B).

The resulting structure shows TFIIH in a conformation that resembles the structure of TFIIH engaged in the transcriptional RNA Pol II preinitiation complex (Pol II–PIC) (*60*–*65*) but bound to XPCc instead of Pol II and general transcription factors (Fig. 3, A to C). Specifically, the conformation of the XPD helicase is identical to our free TFIIH (*28*, *29*) and holo-TFIIH-XPC IEC structures (fig. S14, C and D) and bears close resemblance to the conformation of the yeast homolog RAD3 in the core-TFIIH/Rad4-Rad23-Rad33 DNA opening complex (*48*) and of XPD in the core-TCD structure (fig. S14E) (*47*), except that the latter two complexes lack MAT1 bound at the XPD ARCH domain. The XPD conformation in our holo-TCD complex is distinct from the previously published core-TFIIH-XPC-DNA-XPA (core-TCDA) and core-TFIIH-DNA-XPA (core-TDA) structures (*25*, *47*) (see below).

The helical bundle domain of the CAK subunit MAT1 is visualized bound to the XPD ARCH domain, confirming that the kinase module is present in these complexes (Fig. 3A). The presence of MAT1 has been suggested to enforce an autoinhibited conformation in XPD, in which an autoinhibitory plug (XPD residues 291 to 320) blocks the DNA binding cavity of the helicase (*25*). Whereas density for this autoinhibitory plug is weak in the core-TCD complex lacking the kinase module (*47*), we could visualize interpretable density for this region in our holo-TCD reconstruction, likely due to the presence of MAT1 at the XPD ARCH domain (fig. S14, F and G). Furthermore, the active site and the DNA binding site of XPD are covered by segments of p62, as observed in free TFIIH (*28*, *29*) and the IEC (fig. S14H), indicating that access to the functional centers of XPD is still blocked at this stage of the NER process. Correspondingly, XPD does not interact with any part of the DNA at this stage (Fig. 3A). The observed convergence between the configurations of TFIIH in these transcriptional and DNA repair structures (Fig. 3, A to C) therefore reflects its functional state, with XPB bound to DNA and XPD being inhibited from forming any interactions with DNA.

In comparison with free TFIIH and the holo-TFIIH-XPC IEC, the contacts between the XPB DNA translocase and both MAT1 and XPD in the holo-TCD complex are broken, and the horseshoe-shaped TFIIH core complex adopts a wider conformation (Fig. 3D). These rearrangements are due to a conformational change in XPB. The XPB DRD and the RecA1 domain move away from XPD and toward the DNA, which is likely triggered by XPB binding to dsDNA (Fig. 3D). Transcriptional complexes in which this conformational change within TFIIH is impeded by the presence of a promoter-proximal nucleosome show low transcriptional activity, indicating that this conformational rearrangement in TFIIH is linked to XPB activation (*63*). The conformation of XPB in the holo-TCD complex thus represents an activated state of XPB, similar to previous observations in DNA-bound structures of both NER complexes (*25*, *47*, *48*) and the transcriptional Pol II–PIC (Fig. 3, A to D) (*60*–*62*).

It was previously hypothesized that binding of XPA induces a TFIIH conformation that is incompatible with the MAT1-XPB interaction due to distance constraints, thereby ejecting MAT1 and the CAK from TFIIH and derepressing TFIIH (*25*). However, our structure indicates that the presence of XPA is not required for breakage of the MAT1-XPB contact in NER complexes. Furthermore, breakage of this contact alone is insufficient to induce MAT1 dissociation, which would cause CAK release from core TFIIH and displacement of the autoinhibitory segments of p62 from XPD. This is consistent with the isolation of stable XPD-MAT1 complexes in the absence of other TFIIH subunits (*23*, *24*, *26*) and with biochemical data demonstrating a requirement for ATP for CAK release from TFIIH during NER (*30*). Overall, these observations suggest the existence of additional, structurally uncharacterized, intermediate states between DNA binding and full activation of TFIIH by release of the CAK.

### Binding of XPA incompletely remodels TFIIH

**Fig. 4.**
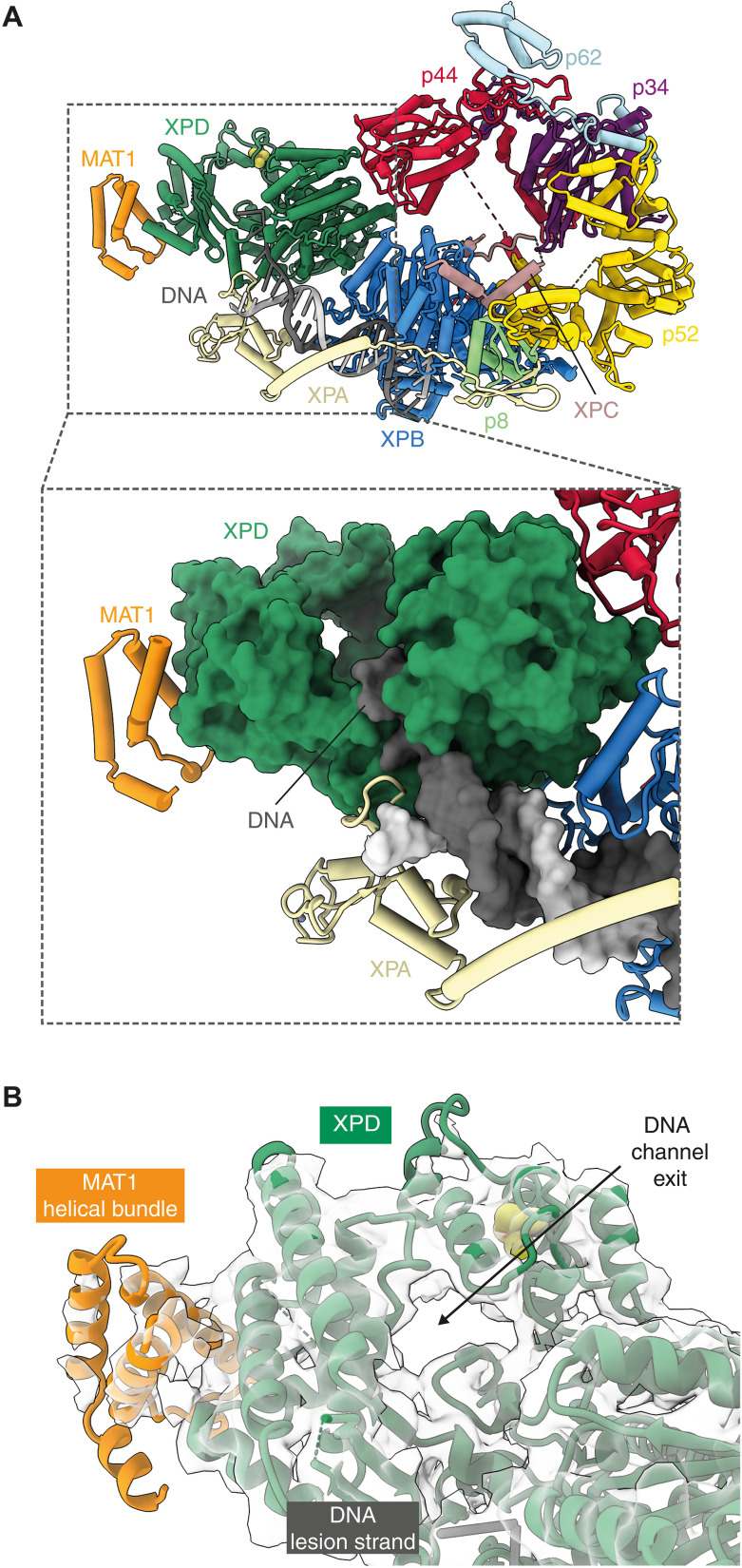
Partial derepression of TFIIH by XPA. (**A**) Cartoon representation of the atomic model of the holo-TCDA complex, colored by subunit with ligands depicted as spheres. Inset: Close-up view of ssDNA (lesion strand: dark gray; nonlesion strand: light gray) interacting with XPD (green), shown as surfaces. (**B**) Density for XPD, MAT1, and the DNA lesion strand of holo-TCDA with an unobstructed DNA exit channel highlighted. The cryo-EM map was low-pass filtered to 4 Å for visualization.

Addition of XPA to the holo-TCD complex results in the visualization of a holo-TCDA complex (Fig. 4A and fig. S15, A and B). The portions of XPC that were bound to the DNA in the holo-TCD complex are no longer visualized, but a C-terminal segment remains bound to the XPB RecA2 domain. This indicates that XPC has been mobilized after XPA binding but remains flexibly tethered to XPB. The DNA in this complex has been modeled to provide the best overall consistency with the pattern of purines and pyrimidines observed in the cryo-EM density. However, the assignment is not unambiguous, and we acknowledge that the DNA might be bound in multiple positions or orientations. We have restricted our interpretation accordingly.

In the holo-TCDA complex, XPD assumes the conformation that has been visualized previously in core-TDA and core-TCDA structures, both of which were assembled in the absence of MAT1 (fig. S15, C and D) (*25*, *47*), and it is starting to engage with ssDNA (Fig. 4A and fig. S15, A, B, and E). We visualize three nucleotides of single-stranded DNA interacting with the XPD RecA2 domain (fig. S15E), although the DNA density does not reach the pore between the XPD FeS and ARCH domains. Consistent with these initial DNA interactions, the density for the p62 segments covering the functional centers of XPD is weak and fragmented in this structure. In comparison with the holo-TCD structure, there is an additional, weak density observed on the XPD RecA2 domain (fig. S15, F and G). This density is incompatible with the p62 conformation observed in the TFIIH-XPC IEC and holo-TCD structures, consistent with a conformational change in p62, although the density segment is too short and fragmented to assign it to a specific sequence (fig. S15, F and G). Overall, this indicates that the p62 segments covering the ATP-binding site and DNA binding groove of XPD have been mobilized and mostly released upon XPA binding, probably due to the XPA-induced conformational change of XPD (Fig. 4A and fig. S15, H and I). These findings are consistent with the previous core-TDA and core-TCDA structures in the absence of the CAK (fig. S15, C and D) (*25*, *47*). However, unexpectedly, the MAT1 helical bundle domain is still visualized bound to the XPD ARCH domain in the holo-TCDA complex (Fig. 4, A and B), indicating that the release of CAK has not been fully completed. Notably, we did not observe any intact XPA-containing complexes without MAT1 in our cryo-EM data. This indicates that the presence of CAK and XPA on TFIIH is not mutually exclusive, which is again consistent with prior biochemical data demonstrating that both the presence of XPA and the availability of ATP—which was absent from our cryo-EM sample—are required for CAK release during NER (*30*). In the absence of ATP, the CAK (including MAT1) is retained on TFIIH, even when XPA is present (*30*), which is consistent with our observation. Although MAT1 is still bound to XPD, density for the XPD autoinhibitory plug is weak, suggesting that the presence of MAT1 alone is not sufficient to keep the plug in its autoinhibitory conformation when XPD starts to undergo the conformational changes, leading to its conformation observed in our structure (fig. S15, H and I). These combined findings suggest that XPA contributes to the remodeling of XPD, thereby rendering its enzymatic active site accessible for nucleotide binding and priming the complex for activation. However, additional ATP-driven conformational changes in XPD or more globally in the assembling preincision complex are likely required to fully dislodge MAT1 from the XPD ARCH domain (*30*), thereby ejecting the CAK from TFIIH during NER, allowing DNA translocation by XPD, and making the ARCH domain available for binding of XPG. The state of TFIIH we observe in the holo-TCDA complex is thus an additional intermediate that is likely to be populated transiently before TFIIH reaches a core-TCDA–like state in which MAT1 is fully removed.

## DISCUSSION

**Fig. 5.**
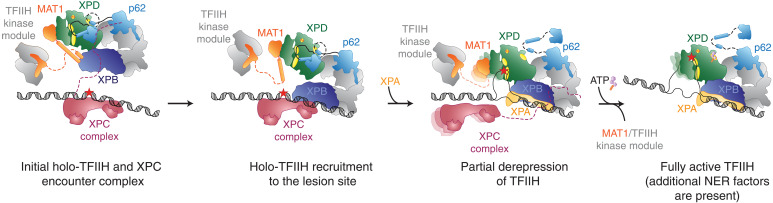
Schematic summary of stepwise derepression of TFIIH in GG-NER. First, holo-TFIIH and XPC form an IEC where XPC recruits TFIIH at the lesion site. Then, TFIIH engages with DNA through its DNA translocase XPB, thereby breaking the MAT1-XPB interaction but preserving the MAT1-XPD interaction, which keeps the complex in an autoinhibited form. Upon XPA binding, XPD undergoes a conformational change that leads to the displacement of XPD-blocking fragments, which partially derepresses TFIIH. At this point, both XPA and MAT1 are bound to the TFIIH core. Availability of ATP is required to fully derepress XPD and release the kinase module from core TFIIH to proceed to DNA unwinding and damage verification.

Here, we determined the high-resolution structures of three intermediates of early stages of human GG-NER using recombinantly expressed, purified components, streptavidin affinity grids, and a biotinylated damaged DNA to assemble these complexes. Our three high-resolution structures reveal how autoinhibited holo-TFIIH, in the presence of the CAK subcomplex, is recruited to DNA lesions and provide mechanistic insight into the structural transitions as NER progresses from DNA-bound XPCc to a state where TFIIH is derepressed and ready to start unwinding DNA (Fig. 5 and table S4). Early in the process, holo-TFIIH and XPC form an IEC where XPC recruits autoinhibited holo-TFIIH to the lesion site by binding to the p62 BSD2 and PH domains. Although the PH domain is flexible in our structure, previous structural studies (*17*) and the domain organization of p62 in our complex indicate that these two p62 domains can interact with their respective binding regions in XPC simultaneously (Figs. 2C and 5). Subsequently, TFIIH engages with the DNA upstream of the lesion through the DNA translocase XPB. Upon DNA binding, XPB undergoes a conformational change that results in the breakage of the MAT1-XPB contact. Meanwhile, the MAT1-XPD interaction is preserved, which keeps the complex in an autoinhibited form, as indicated by the retention of inhibitory p62 segments at important functional sites of XPD (Fig. 5). Accordingly, at this point, XPD is not yet interacting with any parts of the DNA. Upon XPA binding, XPD undergoes a conformational rearrangement that leads to the displacement of the XPD inhibitory p62 fragments, which primes TFIIH for full activation. Notably, both XPA and MAT1 are bound to core TFIIH simultaneously at this stage, and ATP binding or hydrolysis, possibly in conjunction with contributions from other NER factors, is required to fully release the kinase module from core TFIIH and proceed to DNA unwinding and damage verification (*30*). In a cellular environment, other downstream factors of the pathway, such as the two endonucleases XPF-ERCC1 and XPG, are likely to be present to stimulate XPD helicase activity and prepare for dual incision at this stage (Fig. 5). While this manuscript was undergoing peer review, a study detailing the later stages of NER preincision complex assembly was published (*52*). The data in this study are compatible with our findings and suggest a possible involvement of XPC in CAK release.

In contrast to a prior cryo-EM study of the stepwise assembly of the human core-TFIIH-XPC-XPA complex on damaged DNA (*47*), our analysis did not resolve an intermediate in which XPA and the DNA binding domain of XPC are visualized simultaneously. This might suggest that this intermediate is short lived and was stabilized by chemical cross-linking used by Kim *et al.* (*47*). It is possible that short-lived states with low particle populations might not be detected in our affinity-grid–based approach without cross-linking despite the collection of very large datasets. Conversely, the TFIIH-XPC IEC was first visualized using our affinity-grid based approach. This suggests that affinity-grid–based and cross-linking–enabled structural analysis may be complementary techniques that capture overlapping but not necessarily identical states in dynamic molecular processes.

In summary, our cryo-EM structures provide insight into the sequence of events during derepression of TFIIH during the initial stages of NER. This provides mechanistic information about a critical DNA repair pathway that safeguards the genome from UV- and chemically induced damage. In addition, high-resolution structures of NER intermediates may guide the exploration of new therapeutic strategies and the rational design of therapeutics to modulate the activity of the pathway for treatment of human pathologies. The observation that XPD-deficient urothelial cancers are particularly sensitive to base-modifying chemotherapeutics (*66*) suggests that reducing the activity of TFIIH or NER in general may be an avenue toward sensitizing cancer cells to such treatments.

## MATERIALS AND METHODS

### Expression constructs

A list of synthetic DNA molecules used in this study is provided in table S5. Codon-optimized sequences of XPC, hRAD23B, CETN2, and XPA (Twist Bioscience) were amplified from donor plasmids by polymerase chain reaction (PCR). Amplified genes were then cloned into the SspI site of the MacroBac (438-series) vectors using sequence- and ligation-independent cloning (SLIC) (*67*, *68*). XPC was cloned into the 438-C vector [N-terminal His_6_-maltose-binding protein (MBP) with Tobacco Etch Virus (TEV) cleavage site], hRAD23B was cloned into the 438-Sn vector (N-terminal twin Strep tag with TEV cleavage site), CETN2 was cloned into the 438-A vector (untagged), and XPA was cloned into the 438-B vector (N-terminal His_6_ tag with TEV cleavage site). The three genes forming the XPCc were then combined into a single vector for expression by stepwise assembly of the individual cassettes expressing one gene each into multiprotein expression constructs. Assembly was performed using In-Fusion Snap Assembly Master Mix (Takara).

Vectors encoding codon-optimized p62, p44, p34, XPD, and XPB and a p52-p8 fusion protein were ordered from Twist Bioscience or GenScript (XPD and XPB), and coding sequences were amplified by PCR. p62 was synthesized with a C-terminal Twin Strep-His_10_ tag but the His_10_ tag was eventually removed by introducing a stop codon to allow tagging of XPB with a His_6_ tag instead. Similarly, the p52-p8 fusion was later split to be expressed as separate proteins. Amplified genes were cloned into the MacroBac vectors using SLIC (*67*, *68*): XPD, p62, p52, p44, p34, p8, CDK7, cyclin H, and MAT1 were cloned into the 438-A vector (untagged), and XPB was cloned into the 438-B vector (N-terminal His_6_ tag). 438-A-XPD was further modified to include a C-terminal FLAG tag using Gibson assembly methods (*69*). XPB, p62, p52, p44, p34, and p8, as well as CDK7, cyclin H, and MAT1, were combined into two vectors encoding six subunits of the TFIIH seven-core complex (all but XPD) and the CAK subcomplex, respectively, by stepwise assembly using SLIC.

### Baculoviruses and protein expression

For all complexes, multiprotein expression vectors were transformed into EMBacY chemically competent cells, and bacmids (*70*) were isolated by isopropanol precipitation. Sf9 (*Spodoptera frugiperda*) and High Five (*Trichoplusia ni*) cells (both Thermo Fisher Scientific, catalog nos. 11496015 and B85502, respectively) were grown in Sf-900 II SFM medium (Gibco) at 27°C. For generation of recombinant baculoviruses, purified bacmids were transfected into 8 × 10^5^ Sf9 (*S. frugiperda*) insect cells seeded in six-well plates (Thermo Fisher Scientific) using Cellfectin II transfection reagent (Thermo Fisher Scientific). At 3 days posttransfection, cells and supernatant were harvested as the first passage (P1) baculovirus, which was then used to infect 30 ml (5 × 10^5^ cells/ml) of Sf9 cells. At about 4 days postinfection, supernatant was harvested by centrifugation at ×1000*g* for 10 min and was stored at 4°C, supplemented with 2% fetal bovine serum (Gibco).

To express XPCc and XPA, P2 viruses were further amplified to generate P3 virus by infecting 30 ml (5 × 10^5^ cells/ml) of Sf9 cells with 1 ml of P2 virus. The P3 virus was harvested at 3 days postinfection and was used for recombinant protein production in High Five cells or Sf9 cells. To express XPCc, 30 ml of P3 XPCc baculovirus was used to infect 750 ml of High Five cells at a density of 1 × 10^6^ cells/ml. Infected cells were grown for 72 hours, harvested by centrifugation (10 min at ×1000*g*), snap frozen in liquid nitrogen, and stored at −80°C until use. To express XPA, 30 ml of P3 baculovirus was used to infect 1 liter of Sf9 cells at a density of 5 × 10^5^ cells/ml. Cells were harvested 72 hours postinfection by centrifugation (10 min at ×1000*g*), snap frozen in liquid nitrogen, and stored at −80°C until use.

TFIIH core subunits (XPB, XPD, p62, p52, p44, p34, and p8) and CAK (CDK7, cyclin H, and MAT1) were expressed separately in Sf9 cells and combined during protein purification. TFIIH seven cores was expressed by coinfecting 2 liters of Sf9 cells with 13.4 ml of XPB, p62, p52, p44, p34, and p8 combined P2 virus and 26.6 ml of XPD P2 virus. CAK was expressed by infecting 2 liters of Sf9 cells with 20 ml of CAK P2 virus. After 72 hours of incubation, cells expressing TFIIH seven cores and CAK were harvested by centrifugation (10 min at ×1000*g*), snap frozen in liquid nitrogen, and stored at −80°C until use.

### Protein purification

All buffers were filtered and thoroughly degassed by sonication under vacuum for 15 min.

#### Holo-TFIIH

To form the holo-TFIIH complex, insect cell pellets from TFIIH seven-core and CAK-expressing cells were combined and resuspended in TFIIH lysis buffer [40 mM Hepes-KOH (pH 7.9), 250 mM KCl, 2 mM MgCl_2_, 10 mM imidazole, 10% (v/v) glycerol, and 2 mM tris(2-carboxyethyl)phosphine (TCEP)] supplemented with protease inhibitors and deoxyribonuclease I (DNase I; Roche). After lysis by 4 cycles of freeze thaw, the cell lysate was clarified by centrifugation at 18,000 rpm for 30 min at 4°C in a F0650 rotor (Beckman Coulter). Cleared cell lysate was then incubated with preequilibrated Ni-NTA Superflow resin (QIAGEN) for 1 hour at 4°C on a roller shaker. After incubation, Ni-NTA resin was washed with TFIIH wash buffer A [40 mM Hepes-KOH (pH 7.9), 250 mM KCl, 2 mM MgCl_2_, 30 mM imidazole, 10% (v/v) glycerol, and 2 mM TCEP], and protein complexes were eluted with TFIIH elution buffer A [40 mM Hepes-KOH (pH 7.9), 250 mM KCl, 2 mM MgCl_2_, 300 mM imidazole, 10% (v/v) glycerol, and 2 mM TCEP]. Eluted complexes were then incubated with anti-FLAG M2 affinity gel (Sigma-Aldrich) for 1 hour at 4°C on a roller shaker. After incubation, the anti-FLAG M2 affinity gel was washed with TFIIH wash buffer B [40 mM Hepes-KOH (pH 7.9), 250 mM KCl, 2 mM MgCl_2_, 10% (v/v) glycerol, and 2 mM TCEP], and complexes were eluted with wash buffer B supplemented with 100 μg/ml of 3× FLAG peptide (Sigma-Aldrich). Eluted complexes were concentrated using a Vivaspin 6 100-kDa concentrator (Cytiva) at ×1000*g* and injected onto a Superose 6 Increase 10/300 GL column (Cytiva) preequilibrated in TFIIH gel filtration buffer [20 mM Hepes-KOH (pH 7.9), 250 mM KCl, 2 mM MgCl_2_, 10% (v/v) glycerol, and 2 mM TCEP]. Peak fractions (fig. S1, B and D) were pooled and concentrated to 0.7 to 1.7 mg/ml, snap frozen in liquid nitrogen, and stored at −80°C.

#### XPC complex

To purify the XPC-hRAD23B-CETN2 complex, a pellet from 750 ml of High Five cell culture was resuspended in XPC/XPA lysis buffer [40 mM Hepes-KOH (pH 7.9), 250 mM KCl, 2 mM MgCl_2_, 10 mM imidazole, 10% (v/v) glycerol, and 5 mM β-mercaptoethanol] supplemented with protease inhibitors and DNase I (Roche). After lysis by sonication for 5 min (10-s on, 20-s off, and 50% amplitude) on ice, cell debris was pelleted by centrifugation at 18,000 rpm for 30 min at 4°C in a F0650 rotor (Beckman Coulter). Cleared cell lysate was then incubated with preequilibrated Ni-NTA Superflow resin (QIAGEN) for 1 hour at 4°C on a roller shaker. After incubation, Ni-NTA resin was washed with XPC/XPA wash buffer A [40 mM Hepes-KOH (pH 7.9), 250 mM KCl, 2 mM MgCl_2_, 30 mM imidazole, 10% (v/v) glycerol, and 5 mM β-mercaptoethanol], and complexes were eluted with XPC/XPA elution buffer A [40 mM Hepes-KOH (pH 7.9), 250 mM KCl, 2 mM MgCl_2_, 300 mM imidazole, 10% (v/v) glycerol, and 5 mM β-mercaptoethanol]. Eluted complexes were then diluted with low-salt buffer [40 mM Hepes-KOH (pH 7.9), 150 mM KCl, 2 mM MgCl_2_, 10% (v/v) glycerol, and 5 mM β-mercaptoethanol] to achieve a final concentration of 200 mM KCl. Affinity tags were cleaved using His_6_-tagged TEV protease (1:5 TEV:protein complex ratio) at 4°C overnight. Subsequently, tags and TEV protease were removed by reverse-phase nickel affinity chromatography. Fractions containing XPCc were then concentrated by 50-kDa cutoff Amicon Ultra centrifugal filter (Merck) and applied to a Capto HiRes Q 5/50 column (Cytiva) preequilibrated in low-salt buffer. After washing the column with 5 column volumes of low-salt buffer, complexes were eluted with a 0 to 45% gradient of high-salt buffer [40 mM Hepes-KOH (pH 7.9), 1 M KCl, 2 mM MgCl_2_, 10% (v/v) glycerol, and 5 mM β-mercaptoethanol]. Fractions containing XPCc were pooled, concentrated, and applied to a Superdex 200 Increase 10/300 GL column (Cytiva) preequilibrated in XPC/XPA gel filtration buffer [20 mM Hepes-KOH (pH 7.9), 200 mM KCl, 2 mM MgCl_2_, 10% (v/v) glycerol, and 5 mM β-mercaptoethanol]. Peak fractions (fig. S1, B and C) were pooled and concentrated to 2 mg/ml, snap frozen in liquid nitrogen, and stored at −80°C.

#### XPA

A pellet from 1 liter of Sf9 cells was resuspended in 40 ml of XPC/XPA lysis buffer supplemented with protease inhibitors and DNase I. Cells were lysed by sonication on ice, and cell lysate was cleared by centrifugation in a Beckman Coulter JA-25.50 fixed-angle rotor at 18,000 rpm for 30 min at 4°C. The supernatant was incubated with preequilibrated Ni-NTA Superflow resin (QIAGEN) for 1 hour at 4°C on a roller shaker. The resin was then washed three times with XPC/XPA lysis buffer supplemented with 25 mM imidazole and eluted in 3 × 5 ml of XPC/XPA elution buffer A. All three elutions were combined, concentrated, and loaded on a Superdex 200 Increase 10/300 GL column (Cytiva) preequilibrated in XPC/XPA gel filtration buffer for size exclusion chromatography. Peak fractions were analyzed by SDS–polyacrylamide gel electrophoresis (SDS-PAGE). Selected fractions were pooled, concentrated to 2 mg/ml, snap frozen in liquid nitrogen, and stored at −80°C (fig. S1, B and E).

### DNA substrate preparation

Lesion-containing DNA strand (5′-CCCATTCCAACCATCCCACTACC-iCy5-TCCATCTTCACTCAACTATCACCC-3′) and biotinylated DNA strand (5′-/5Biosg/AGAGAAGGAGAGGCAAGGAAGGGTGATAGTTGAGTGAAGATGGACAGGTAGTGGGATGGTTGGAATGGG-3′) were ordered from Integrated DNA Technologies (see table S5) and dissolved to a final concentration of 100 μM. Biotinylated lesion-containing DNA was annealed by mixing equimolar quantities of each strand in water, heating the reaction for 5 min at 95°C, and gradually (−1°C/min) cooling the reaction to 4°C in a C1000 Touch Thermal Cycler (Bio-Rad).

### Electrophoretic mobility shift assay

Lesion-containing dsDNA (0.5 μM) was incubated with equimolar quantities of XPCc, XPA, and holo-TFIIH in 20 μl of reaction buffer [25 mM tris HCl (pH 8.0), 100 mM KCl, 5 mM dithiothreitol, 7 mM MgCl_2_, and 8% (v/v) glycerol]. Each reaction was incubated for 30 min at 30°C. Complexes were resolved by gel electrophoresis on a NuPAGE Tris-Acetate 7% gel. Gels were initially scanned on a Typhoon FLA 9500 imager to detect the iCy5-labeled DNA and subsequently Coomassie stained (InstantBlue Coomassie Protein Stain, Abcam) and imaged on a ChemiDoc Imaging System (BioRad).

### Cryo-EM sample preparation

Quantifoil Au R 2/2 streptavidin affinity grids were prepared in house following established protocols (*54*). Holo-TFIIH-XPC-DNA complexes were assembled by incubating 250 nM biotinylated lesion-containing DNA with XPCc and holo-TFIIH at a 1:1:1 ratio in sample buffer [50 mM Hepes-KOH (pH 7.5), 150 mM KCl, and 0.5 mM TCEP] for 10 min at 20°C. Holo-TFIIH-XPC-XPA-DNA complexes were assembled with addition of XPA at a 1:1:1:1 ratio. A total of 4 μl of the complex was then incubated on streptavidin affinity grids (prehydrated in sample buffer for 10 min) for 1 min in a humidity chamber.

After incubation, grids were washed four times with 10 μl of freezing buffer [50 mM Hepes (pH 7.5), 150 mM KCl, 0.5 mM TCEP, 3% trehalose, and 0.01% NP-40] to remove unbound complexes. After the washes, excess liquid was blotted away by touching Whatman filter paper to the side of the grid, and 4 μl of freezing buffer was immediately applied. Grids were then transferred to a Leica EM GP2 plunge freezer, blotted for 5 s at 20°C and 95% humidity, and then plunged into liquid ethane.

### Data acquisition

Grids were initially screened on a 200-kV Glacios cryo–transmission electron microscope (cryo-TEM) equipped with a Falcon 4i direct electron detector (Thermo Fisher Scientific) operating in counting mode behind a Selectris energy filter (operated using a slit width of 10 eV). Two grids were chosen for in-house data collection, as judged by ice thickness and particle distribution. Data were collected semiautomatically using EPU (Thermo Fisher Scientific) at a pixel size of 0.7 Å, with a total exposure of 60 electrons/Å^2^ and a defocus range of −0.7 to −2.5 μm. Approximately 5000 to 10,000 movies for each grid were collected and processed independently. Electron-event representation (EER)-format movies were motion corrected using cryoSPARC live to assess data quality (*56*). The background streptavidin lattice was subtracted using MATLAB scripts (*54*). The subtracted and aligned movies were then reimported into cryoSPARC v4.6.2 to fit the contrast transfer function (CTF) of each micrograph. Particles were picked using blob picker (circular/elliptical blob; 140- to 240-Å diameter), and particle orientation distribution was assessed by 2D classification.

For high-resolution structure determination, selected grids were loaded into a Titan Krios G3i cryo-TEM (Thermo Fisher Scientific) operated at 300-kV acceleration voltage and equipped with a K3 direct electron detector and a BioQuantum energy filter (both Gatan Inc.). Data were collected in TIFF format at a ×165,000 nominal magnification in counting mode for holo-TCDA grid-1 and in super-resolution mode for holo-TCDA grid-2 and the holo-TCD grid, resulting in a pixel size of 1.34 and 0.67 Å per pixel after 2× hardware binning, respectively, with a total exposure of 60 electrons/Å^2^ and a defocus range of −0.7 to −2.5 μm. Grid squares were selected manually, and holes for data acquisition were automatically detected and selected using appropriate relative ice thickness filters in EPU (Thermo Fisher Scientific) for holo-TCD and holo-TCDA complexes. In total, 50,841 movies were collected from one holo-TCD grid, and 87,957 movies were collected from two holo-TCDA grids (43,357 movies from grid-1 and 44,600 movies from grid-2).

### Image processing

#### General image processing strategy

For all structures, cryo-EM data were initially preprocessed in cryoSPARC live and cryoSPARC v4.6.2 (*56*). The best particles were then transferred to RELION 5.0 beta or RELION 5.0.0 (*58*) for further classification, 3D reconstruction, and final refinement. All refinements were done using BLUSH regularization implemented in RELION 5.0 beta or RELION 5.0.0 (*58*). For all maps, resolutions are reported using the Fourier shell correlation (FSC) at the 0.143 cutoff after gold-standard refinement in RELION (*71*, *72*).

#### Holo-TFIIH, XPCc, and DNA grids: Initial reconstructions of the holo-TFIIH-XPC IEC and the holo-TCD complex

A schematic outline of the data processing pipeline used to obtain initial reconstructions of the holo-TFIIH-XPC-IEC and holo-TCD complex is provided in fig. S4. TIFF format movies were motion corrected with 2× binning in cryoSPARC live. The CTF of each motion-corrected micrograph was fitted in cryoSPARC live (*56*). To facilitate data processing, data collected on holo-TCD–containing grids were split into three subdatasets (13,511, 18,663, and 18,667). For each subdataset, total beam-induced specimen motion, in-frame motion, relative ice thickness, and the quality of CTF fitting were inspected to remove poor-quality micrographs, resulting in 11,631, 13,292, and 14,699 micrographs being accepted for each subdataset. The streptavidin lattice signal was then subtracted from motion-corrected micrographs using previously published scripts (*54*). The motion-corrected, subtracted movies were then reimported to cryoSPARC v4.6.2 for further processing.

For each subdataset, particles were initially picked using blob picker (circular/elliptical blob; 140- to 240-Å diameter), extracted using a box size of 280-pixel edge length, binned to 140-pixel edge length, and subjected to several rounds of 2D classification using a batch size of 400 particles and 50 classes for 50 iterations. Selected 2D averages were used to train Topaz (*73*) from a smaller subset of micrographs, and the generated Topaz model was then applied to select particles from the entire subdataset. Particles picked by Topaz were then extracted as with blob-picked particles and subjected to several rounds of 2D classification using a batch size of 400 particles and 50 classes for 50 iterations. Particles picked by blob picking and particles picked by Topaz were combined, and duplicates were removed. Selected particles for each subdataset were combined, resulting in 1,123,597 particles retained [particle set (i)].

Selected particles [particle set (i)] were subjected to two rounds of 2D classification using 50 classes and a batch size of 400 particles for 50 iterations. Selected particles were then used as an input for heterogeneous refinement, using previously published maps as initial references [EMD-27996 (*47*) and EMD-0452 (*28*)]. Selected particles were then subjected to two more rounds of heterogeneous refinement and one round of ab initio reconstruction using two classes, retaining 122,824 particles. Selected particles were then 2D classified into 50 classes using a batch size of 400 particles and 50 iterations, and the best particles were used to train Topaz using a small subset of micrographs. The generated Topaz model was then applied to the entire set of micrographs collected on grid-1, resulting in 528,447 particles extracted [particle set (ii)]. Particles picked by Topaz [particle set (ii)] and initial particles selected from grid-1 [particle set (i)] were then combined, and duplicates were removed, resulting in 1,504,870 particles retained. Selected particles were subjected to 2D classification and five rounds of heterogeneous refinement, retaining 522,199 particles. Particles were then reextracted without binning using a box size of 280 × 280 pixels and subjected to two more rounds of heterogeneous refinement. Selected particles (223,662 particles for holo-TFIIH-XPC IEC and 257,448 particles for holo-TCD) were then subjected to homogeneous refinement, yielding a 3.5-Å resolution reconstruction of the TFIIH-XPC IEC [map (i)] and a 3.7-Å resolution reconstruction of the holo-TCD complex [map (ii); see fig. S4].

#### Holo-TFIIH, XPCc, XPA, and DNA grids

A schematic outline of the data processing pipeline used to obtain high-resolution reconstructions of the holo-TFIIH-XPC-IEC, holo-TCD, and holo-TCDA complexes is provided in figs. S6 and S7. TIFF format movies were motion corrected in cryoSPARC live (no binning for grid-1 and 2× binning for grid-2). The CTF of each motion-corrected micrograph was fitted in cryoSPARC live. To ease data processing, data collected on each grid were split into two subdatasets (21,680 and 21,677 movies for grid-1 and 27,608 and 16,992 movies for grid-2). For each subdataset, total beam-induced specimen motion, in-frame motion, relative ice thickness, and the quality of CTF fitting were inspected to remove poor-quality micrographs resulting in 19,520 and 19,456 micrographs being accepted for the subdatasets of grid-1 and 25,390 and 15,910 micrographs being accepted for the subdatasets of grid-2. The streptavidin lattice signal was subtracted from the motion-corrected micrographs using previously published MATLAB scripts (*54*). The motion-corrected, subtracted movies were then reimported to cryoSPARC v4.6.2 for further processing.

For each subdataset, particles were initially picked using blob picker (circular/elliptical blob; 140- to 240-Å diameter), extracted and binned to a box size of 140 × 140 pixels, and subjected to several rounds of 2D classification using a batch size of 400 particles and 50 classes for 50 iterations. Selected particles for each subdataset were combined, resulting in 1,211,039 particles retained for grid-1 and 1,416,578 particles retained for grid-2.

#### Holo-TCDA complex: Final reconstruction

Selected particles from grid-1 (1,221,039 particles) were used as input for heterogeneous refinement, using the holo-TFIIH-XPC IEC and holo-TCD initial maps [map (i) and map (ii); see fig. S4] and a previously published reconstruction of core-TCDA [EMD-27998 (*47*)] as initial references, resulting in 160,563 holo-TCDA particles retained. Selected particles were then subjected to four iterative rounds of heterogeneous refinement, resulting in 53,295 particles retained. A total of 52,693 particles was reextracted without binning using a box size of 280 × 280 pixels and classified into 50 2D class averages using a batch size of 400 particles for 50 iterations. A total of 38,543 particles was selected to train Topaz on a smaller subset of micrographs, and the generated Topaz model was then applied to the entire dataset. Particles picked by Topaz were then extracted using a box size of 140 × 140 pixels [2× binning; particle set (ii); fig. S6]. Particles picked by Topaz [particle set (ii)] and intact particles from the first round of heterogeneous refinement [particle set (i)] were combined and subjected to three rounds of 2D classification using a batch size of 400 particles and 50 classes for 50 iterations, resulting in 1,762,272 particles retained. Selected particles were then reextracted without binning using a box size of 280 × 280 pixels and used as an input for two iterative rounds of heterogeneous refinement. A total of 88,912 particles representing holo-TCDA was retained and subjected to homogeneous refinement, yielding a 4.2-Å reconstruction [particle set (v)].

Selected particles from grid-2 (1,416,578 particles) were used as an input for heterogeneous refinement, using the holo-TFIIH-XPC IEC and holo-TCD maps [map (i) and map (ii); see fig. S4] and a previously published reconstruction of core-TCDA [EMD-27998 (*47*)] as initial references, resulting in 135,235 particles representing the holo-TCDA complex retained. Selected particles were then subjected to four iterative rounds of heterogeneous refinement, resulting in 36,940 particles retained. Selected particles were then classified into 50 2D class averages using a batch size of 400 particles for 50 iterations. A total of 19,419 particles was selected to train Topaz on a smaller subset of micrographs (fig. S6), and the generated Topaz model was then applied to the entire dataset. Particles picked by Topaz were subjected to two rounds of 2D classification using a batch size of 400 particles and 50 classes for 50 iterations, resulting in 211,725 particles retained. Particles were then reextracted without binning using a box size of 280 × 280 pixels [particle set (iv); fig. S6] and combined with the best particles from the first round of heterogeneous refinement [particle set (iii)] after removing duplicates. Retained particles (1,381,311 particles) were used as an input for two iterative rounds of heterogeneous refinement. A total of 89,285 particles representing holo-TCDA was retained and subjected to homogeneous refinement, yielding a 4.3-Å resolution reconstruction [particle set (vi); fig. S6].

Selected particles from both grids [particle set (v) for grid-1 and particle set (vi) for grid-2; fig. S6] were then combined and used as an input for heterogeneous refinement, retaining 139,417 particles. To improve map quality, retained particles were then converted to *.star files suitable to use in RELION 5.0.0 using scripts contained in the PYEM package (*74*). Motion-corrected and subtracted micrographs were imported to RELION 5.0.0, and selected particles were reextracted using a box size of 280 × 280 pixels based on the coordinate information from imported particles from cryoSPARC v4.6.2. Extracted particles were then subjected to masked 3D refinement with BLUSH regularization, yielding a 3.7-Å resolution reconstruction. To further improve map quality around the XPD-MAT1 region, particles were classified into two classes by focused 3D classification (regularization parameter τ = 20). A total of 80,158 particles was retained and yielded a 3.8-Å resolution reconstruction with better density in the XPD-MAT1 region. Particles were then further classified into two classes by 3D classification without alignment (regularization parameter τ = 20), retaining 43,458 particles. To further improve the map quality, particles were then subjected to CTF refinement (beam tilt, trefoil, and fourth-order aberrations) (*75*), 3D autorefined, and postprocessed using a user-defined B-factor (−20 Å^−2^), yielding a 3.6-Å resolution reconstruction of the holo-TCDA complex.

#### Holo-TFIIH-XPC IEC and holo-TCD complex: Final reconstructions

To obtain reconstructions for the holo-TFIIH-XPC IEC and the holo-TCD complex, particles were initially picked as described above. Selected particles from both grids [particle sets (a) and (b); see fig. S6] were combined (fig. S7) and subjected to heterogeneous refinement, resulting in 1,176,441 particles retained for holo-TFIIH-XPC IEC and 867,905 particles retained for the holo-TCD complex (fig. S7). For the holo-TFIIH-XPC IEC, particles were subjected to another round of heterogeneous refinement to further clean up the dataset, retaining 1,086,149 particles. Selected particles were then converted to *.star files suitable to use in RELION 5.0.0 using scripts contained in the PYEM package (*74*). Motion-corrected and subtracted micrographs were imported to RELION 5.0.0, selected particles were reextracted using a box size of 280 × 280 pixels and the coordinate information from imported particles from cryoSPARC v4.6.2. Extracted particles were then subjected to masked 3D refinement with BLUSH regularization, yielding a 3.1-Å resolution reconstruction. To further improve map quality around the MAT1/XPD/XPB region (see mask in fig. S7), particles were classified into eight classes by focused 3D classification (regularization parameter τ = 20). A total of 359,288 particles was selected and yielded a 3.3-Å resolution reconstruction with better density in the MAT1/XPD/XPB region. Particles were then further classified into eight classes by focused 3D classification (regularization parameter τ = 20) using a mask encompassing the region around p52 and p8, retaining 323,811 particles. To further improve the map quality, particles were then subjected to CTF refinement (beam tilt, trefoil, and fourth-order aberrations) (*75*). Selected CTF-refined particles were refined and postprocessed using a user-defined B-factor (−10 Å^−2^), yielding a 3.3-Å resolution reconstruction of the holo-TFIIH-XPC IEC (fig. S7).

To improve map quality around the DNA-XPC region of the holo-TCD complex, selected particles (867,905 particles; fig. S7) were converted to *.star files suitable to use in RELION 5.0.0 using scripts contained in the PYEM package (*74*). Motion-corrected and subtracted micrographs were imported to RELION 5.0.0, and selected particles were reextracted using a box size of 280 × 280 pixels and the coordinate information from imported particles from cryoSPARC v4.6.2. Extracted particles were then subjected to masked 3D refinement with BLUSH regularization (*58*), yielding a 3.5-Å resolution reconstruction. Particles were initially classified into four classes by focused 3D classification using a mask encompassing the XPD-MAT1 area (regularization parameter τ = 16). After classification, 562,192 particles were retained and yielded a 3.5-Å resolution reconstruction. To further improve map quality, particles were further classified into six classes by focused 3D classification using a mask encompassing the XPC-DNA area (regularization parameter τ = 16), retaining 166,008 particles with improved density on that region. Selected particles were then subjected to masked 3D autorefinement with BLUSH regularization enabled, yielding a 3.9-Å resolution reconstruction of the holo-TCD complex (consensus map; fig. S7).

Multibody refinement (*59*) was used to improve the overall quality of the map, specifically of the DNA and XPC region which appeared to be mobile, as seen during 3D classification (fig. S13). The map was divided into three overlapping bodies using three masks and subjected to multibody refinement: The N-terminal half of p52, p34, p44, p62, XPD, and MAT1 formed body 1; XPB, p34, p52, p8, CETN2, prelesion DNA, and the XPC BH3 and helical domains formed body 2; and XPB RecA1 and RecA2, p62 BSD2, XPC, hRAD23B, CETN2, and the remainder of the DNA formed body 3 (fig. S13A). The resulting reconstruction revealed substantial improvement in the map quality around the XPC and DNA region and substantially improved resolution for core-TFIIH regions in bodies 1 and 2 (fig. S13, D to G). Each body was then postprocessed with a user-defined B-factor (−40, −40, and −180 Å^−2^, respectively), yielding a 3.5-Å resolution reconstruction for body 1, 3.7 Å for body 2, and 4.6 Å for body 3 (fig. S13B and table S2).

### Model building and refinement

#### Holo-TFIIH-XPC initial encounter complex

The structure of holo-TFIIH [PDB 6NMI (*28*)] was docked into the 3.3-Å resolution cryo-EM map of the holo-TFIIH-XPC IEC and used as the basis for atomic model building in Coot (*76*). The quality of our holo-TFIIH-XPC IEC reconstruction allowed us to build the XPD autoinhibitory plug, blocking its DNA binding cavity (residues 291 to 321), and some unassigned regions of p62 that were previously built as a poly-alanine model, such as residues 161 to 176, which connect the BSD1 and BSD2 domains, and residues 243 to 283, which include a loop that is inserted into the DNA binding cavity of XPD. After building all the subunits of holo-TFIIH, there was a small segment of extra density close to the BSD2 domain of p62. Comparison with a predicted structure of the holo-TCD complex, obtained using the AlphaFold 3 web server (*77*), including all protein subunits visualized in the holo-TCD reconstruction (UniProt IDs used: XPB, P19447; XPD, P18074; p62, P32780; p52, Q92759; p44, Q13888; p34, Q13889; p8, Q6ZYL4; MAT1, P51948 residues 64 to 151; XPC, Q01831 residues 60 to 940; CETN2, P41208; hRAD23B, P54727) and the double-stranded region of our DNA construct with paired bases replacing the iCy5, allowed us to assign the density to a short fragment of XPC (residues 162 to 167). The resulting coordinate model was refined against the final 3.3-Å resolution reconstruction of the holo-TFIIH-XPC IEC using the real-space refinement program in PHENIX (*78*). Regions with poor density in the final cryo-EM reconstruction were additionally restrained by reference restraints: The N-terminal RING domain of MAT1 was restrained to the holo-TFIIH structure (PDB 6NMI) (*28*), the p62 helical bundle and BSD1 domain were restrained to an AlphaFold 3 model (*77*), and p8 and the C-terminal domain of p52 were restrained to the holo-TCD model, which had better density for this region.

#### Holo-TCD complex

Protein chains from our holo-TFIIH-XPC IEC model (XPC, p62, p44, and MAT1) were combined with chains from a core-TCD model produced using the AlphaFold 3 web server (*77*) (XPB, p52, p34, p8, XPC, hRAD23B, and CETN2) and an iCy5-containing DNA, which was produced by mutation from the core-TCD model (*47*) to match our construct sequence. The starting model was docked into relevant maps produced by multibody refinement (*59*) of the holo-TCD complex and used as the basis for atomic model building in Coot (*76*). Body 1 was used to build XPD, p62, p44, and MAT1; body 2 was used to build XPB, p52, p34, p8, C-terminal regions of XPC (residues 889 to 916 and 930 to 940), and DNA proximal to the core of the complex; body 3 was used to build the remainder of XPC, hRAD23B, CETN2, and DNA distal to the core complex. Comparison of body 1, centered on XPD (fig. S13), with interactions predicted in the AlphaFold 3–derived model, allowed us to assign density to the XPC residues 162 to 167, which interact with the BSD2 domain of p62 (in agreement with and providing further support to our interpretation of the holo-TFIIH-XPCc-IEC), as well as assigning the sequence register to residues of p62 positioned toward the C terminus from the loop in the XPD DNA binding cavity (residues 282 to 286). Body 2, centered on XPB, allowed us to build the previously unmodeled C terminus of XPB (residues 772 to 780), which we then used to assign equivalent density in the holo-TFIIH-XPC IEC and holo-TCDA maps. Using body 3, centered on XPC-DNA, we were able to assign density to displaced nucleotides from our damaged DNA mimic and stabilizing residues from XPC. We then produced a composite map of the three bodies using ChimeraX (*79*) and used this for real-space refinement in PHENIX (*78*), with a 3.3-Å resolution cutoff determined from composites of each set of half maps using RELION postprocessing (fig. S13B) (*80*). MAT1 and CETN2 were restrained to the holo-TFIIH structure (PDB 6NMI) and the x-ray crystal structure of CETN2 bound to an XPC peptide [PDB 2OBH (*81*)], respectively. This refined multibody model was then used for real-space refinement in PHENIX in the 3.9-Å resolution holo-TCD consensus map, with the DNA chains restrained to the holo-TCD multibody model and MAT1 and CETN2 restrained as above.

#### Holo-TCDA complex

The structure of core-TCDA [PDB 8EBU (*47*)] and residues 1 to 150 of MAT1 from an AlphaFold 3 model (*77*) of core-TCDA-MAT1 were docked into the cryo-EM map of the holo-TCDA complex and used as the basis for atomic model building in Coot (*76*). The DNA sequence register was assigned guided by the best overall consistency with our cryo-EM density and by comparison to the core-TCDA structure [PDB 8EBU (*47*)]. As indicated in the Results section, the DNA sequence register assignment was not unambiguous, and it is possible that the complex binds the model DNA in multiple sequence registers and/or orientations. The resulting coordinate model was refined against the final 3.6 Å-resolution reconstruction of the holo-TCDA complex using the real-space refinement program in PHENIX (*78*). Some regions of the final cryo-EM map had poor density, and coordinates on those regions were additionally restrained by reference restraints. The XPD FeS and ARCH domains and the MAT1 helical bundle were restrained to the holo-TFIIH-XPC IEC model. The p62 helical bundle was restrained to an AlphaFold 3 model (*77*) of the p62 helical bundle and the BSD1 domain. The C-terminal region of p52, which interacts with p8, was restrained to the holo-TCD model, which had higher quality density in this region. The C-terminal region of XPA was restrained to the structure of core-TCDA [PDB 8EBU (*47*)].

### Visualization of molecular models and creation of figures

Molecular models and cryo-EM maps were visualized in UCSF Chimera (*82*), UCSF ChimeraX (*79*, *83*), and PyMOL (The PyMOL Molecular Graphics System, Schrödinger, LLC) for analysis, interpretation, and figure preparation.
