# Supplementary material for: Visualization of stepwise derepression of TFIIH in global genome nucleotide excision repair
Source: Sci Adv. 2026 Jul 23;12(30):eaeb3506. doi: 10.1126/sciadv.aeb3506 (PMC13394385; doi:10.1126/sciadv.aeb3506)
Supplement: Supplementary file 1 — Figs. S1 to S15 Tables S1 to S4 Legend for table S5 Legend for data S1 [file sciadv.aeb3506_sm.pdf]

Science Advances
AAAS

Supplementary Materials for

Visualization of stepwise derepression of TFIIH in global genome nucleotide excision repair

Natàlia de Martín Garrido *et al.*

Corresponding author: Basil J. Greber, basil.greber@icr.ac.uk

Sci. Adv. **12**, eaeb3506 (2026)
DOI: 10.1126/sciadv.aeb3506

The PDF file includes:

Figs. S1 to S15
Tables S1 to S4
Legend for table S5
Legend for data S1

Other Supplementary Material for this manuscript includes the following:

Table S5
Data S1

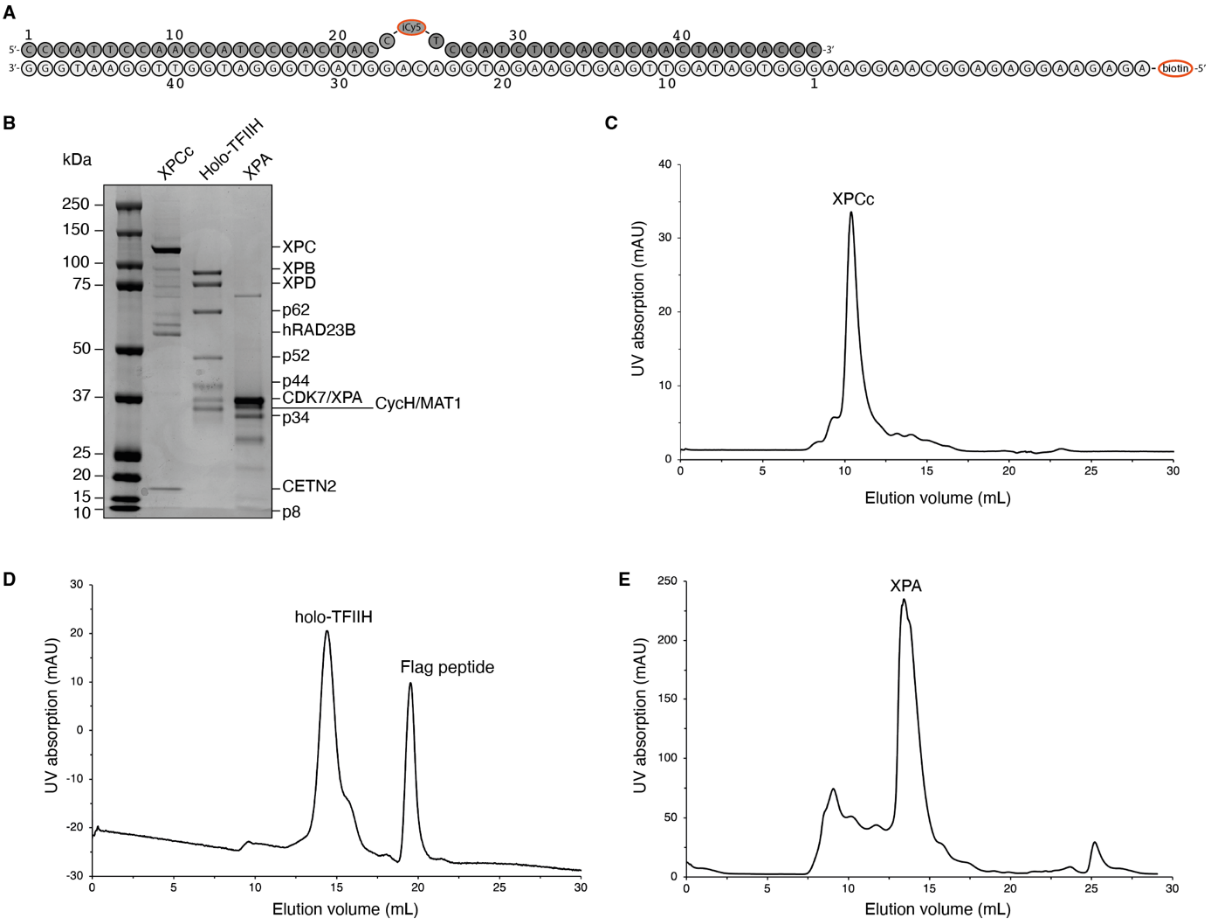

Figure S1. Schematic of biotinylated DNA and purification of NER components. (**A**) Schematic of biotinylated DNA with iCy5 building block, used to assemble and tether NER complexes to cryo-EM grids. (**B**) SDS-PAGE analysis of purified NER complexes and factors visualised by Coomassie staining. Protein bands are labelled. Due to its small size, p8 stains poorly and is not clearly visualised in the gel. However, both p8 and p34 are clearly visualised in our cryo-EM maps, confirming their presence in our purified complex. (**C**-**E**) Size-exclusion chromatography elution profiles of XPCc (C), holo-TFIIH (D), and XPA (E).

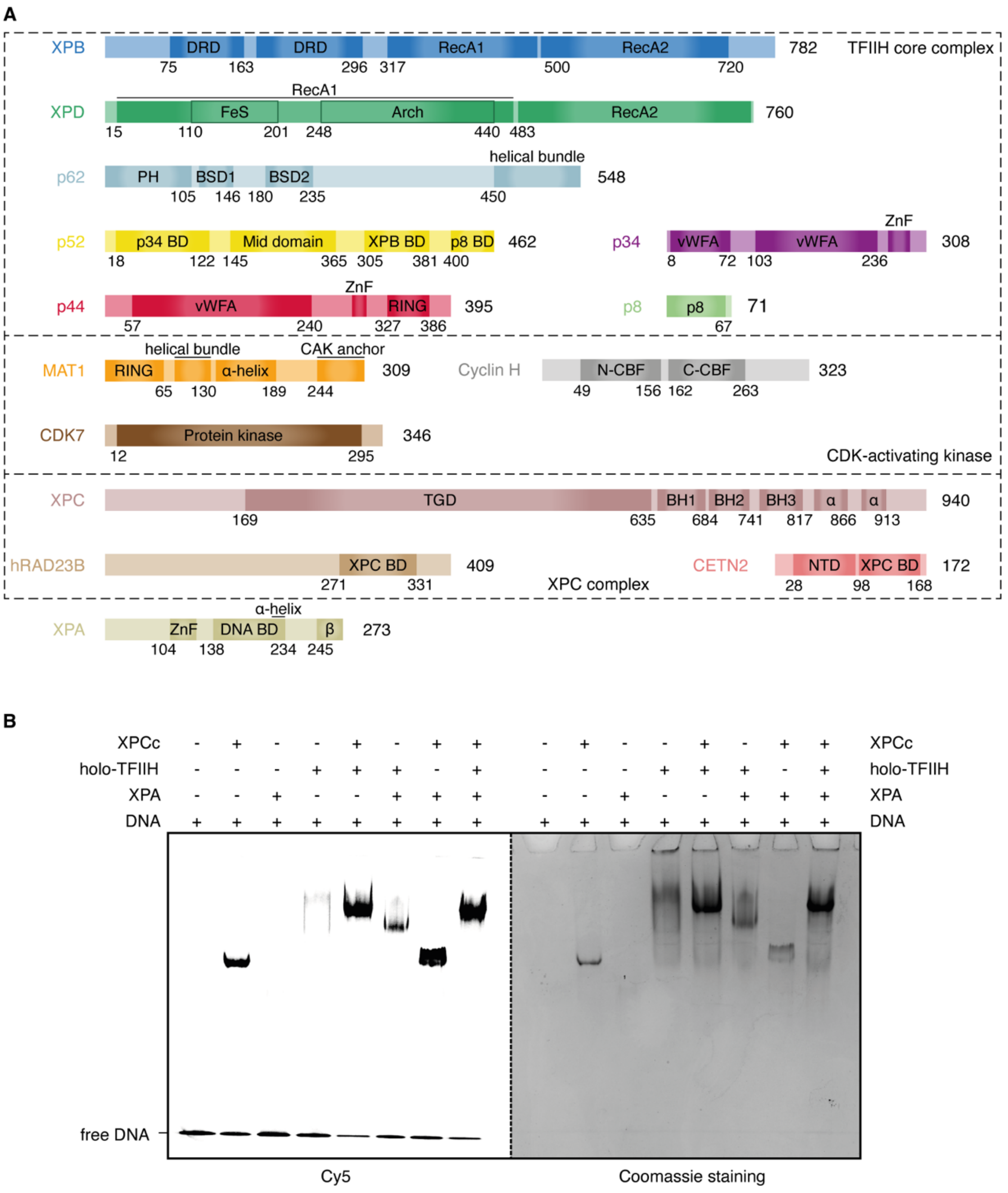

Figure S2. Domain organisation of components of holo-TFIIH, the XPC complex and XPA, and binding to a biotinylated DNA containing an iCy5 lesion mimic. (**A**) Each protein is colour-coded as in Figure 1. Rectangles represent structural domains and are annotated. Abbreviations: DRD, damage recognition domain; FeS, iron-sulfur cluster domain; PH, Pleckstrin homology; BD, binding domain; vWFA, von Willebrand factor type A; RING, really interesting new gene; CBF, cyclin box fold; TGD, transglutaminase-homology domain; BH, β-hairpin domain; NTD, N-terminal domain; ZnF, zinc finger domain. Multi-protein complexed are indicated by dashed boxes. (**B**) Analysis of binding of purified NER components to biotinylated damaged DNA by EMSA, visualised by iCy5 fluorescence (left) and Coomassie staining (right).

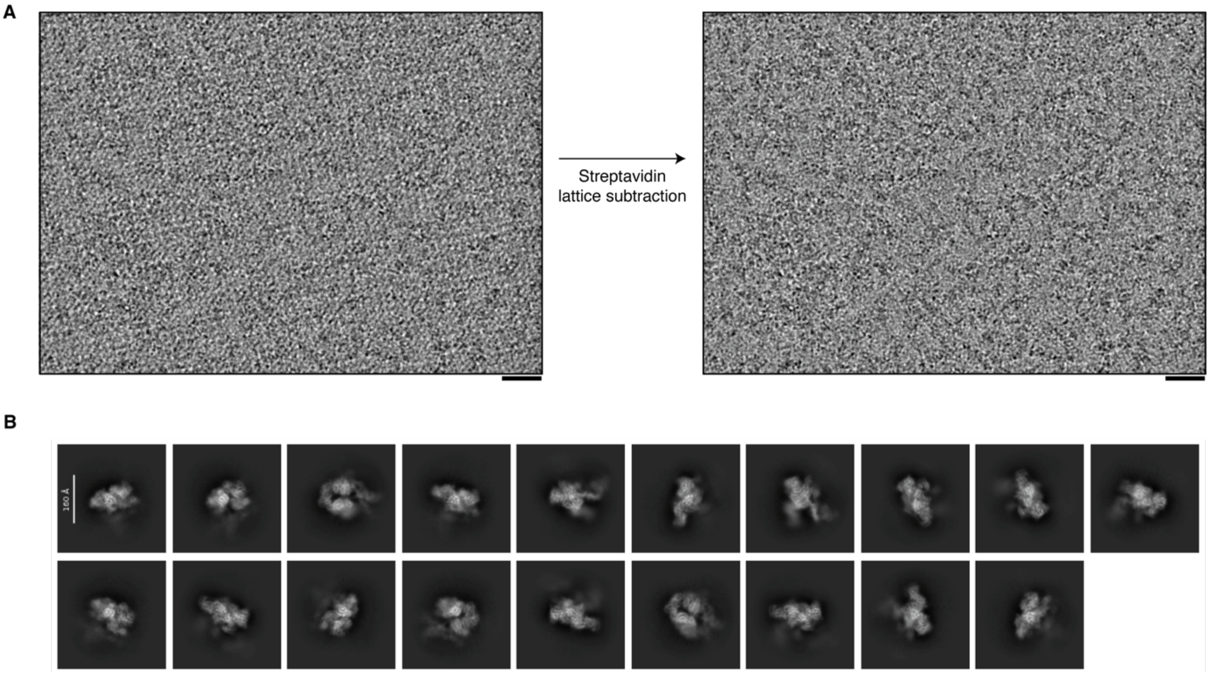

Figure S3. Representative micrographs and 2D classes from holo-TFIIH-XPCc-DNA cryo-EM grids. (**A**) Representative micrographs before and after streptavidin crystal lattice subtraction. Scale bar: 30 nm. Micrographs are low-pass filtered to 10 Å. (**B**) Representative 2D classes of a mixture of holo-TFIIH-XPC IEC and holo-TCD complexes after blob picking.

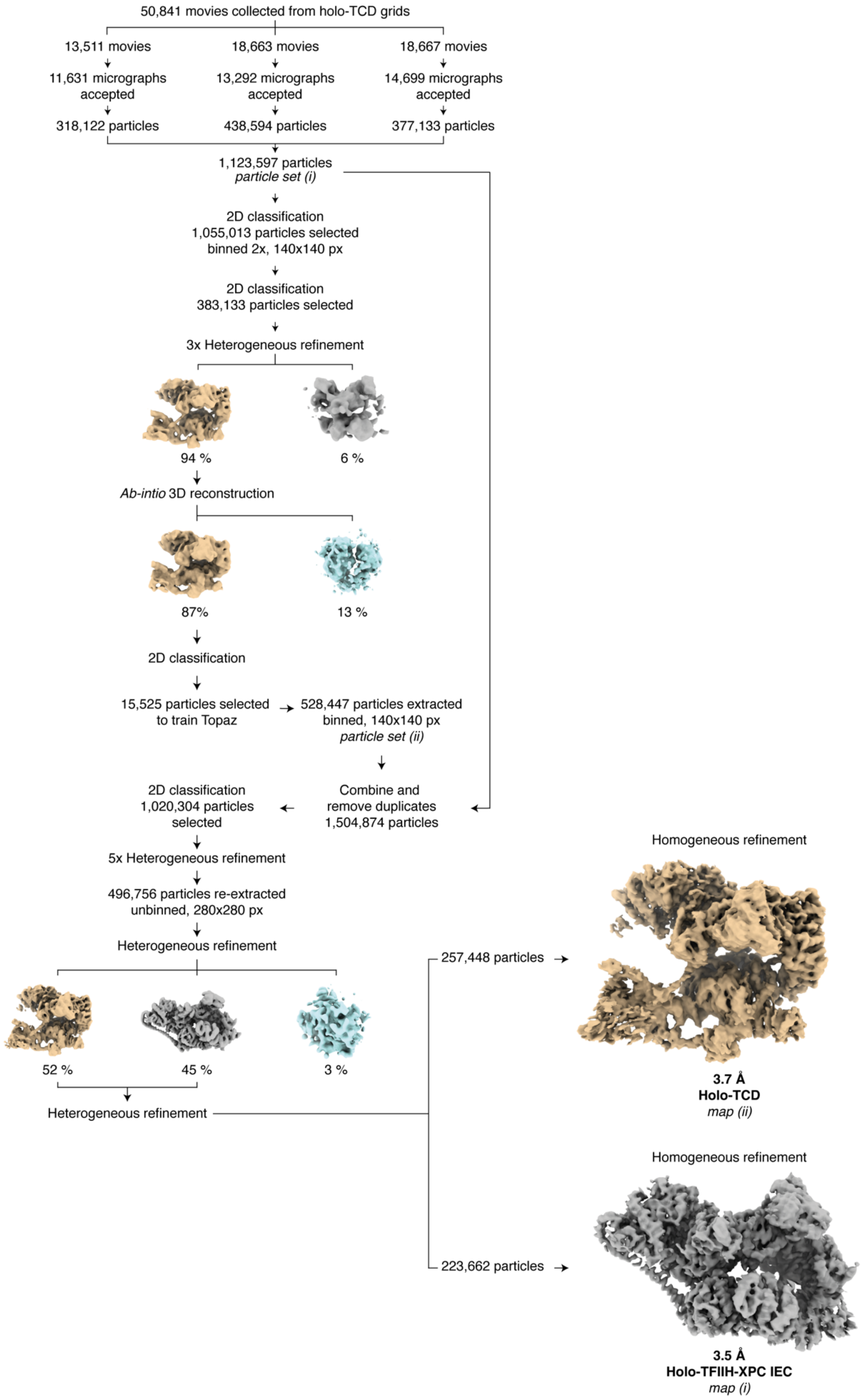

Figure S4. Cryo-EM data processing pipeline for initial reconstructions of the holo-TFIIH-XPC IEC and the holo-TCD complex from holo-TFIIH-XPCc-DNA grids. Pipeline

shows the processing strategy used to obtain initial reconstructions of the holo-TFIIH-XPC IEC and holo-TCD complex. Maps discussed in the main text are labelled in bold. Particle distributions from classification are shown as percentages of total output particles.

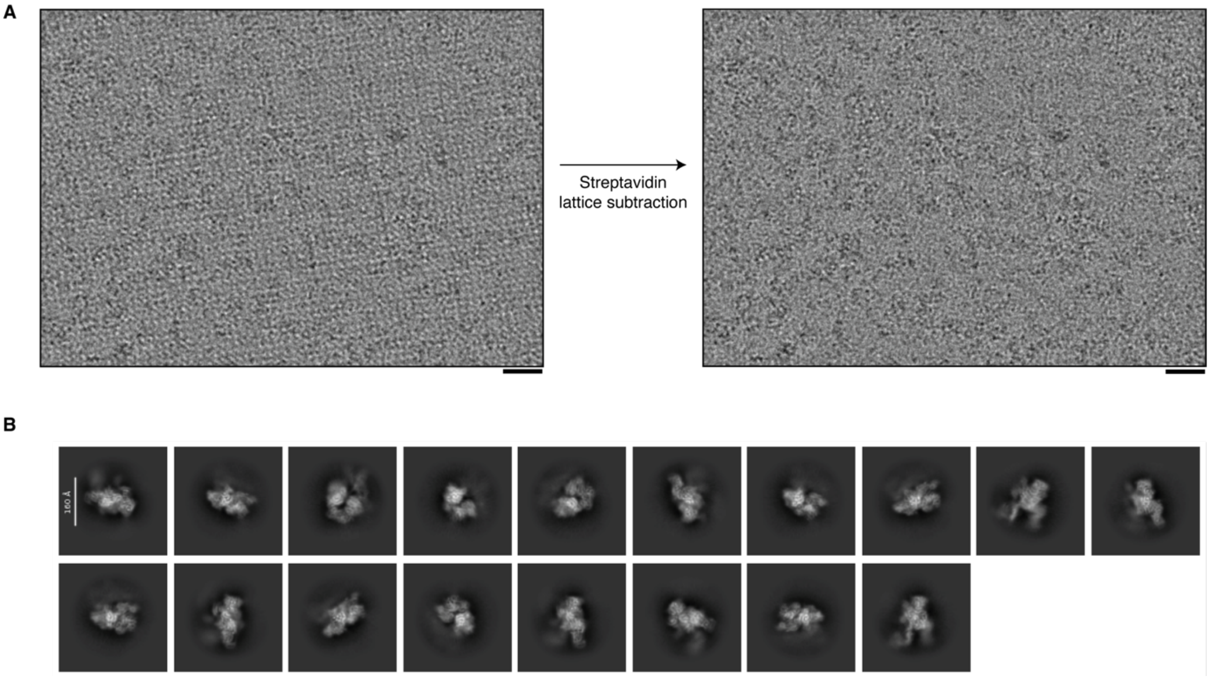

Figure S5. Representative micrographs and 2D classes from holo-TFIIH-XPCc-DNA-XPA cryo-EM grids. (**A**) Representative micrographs before and after streptavidin crystal lattice subtraction. Scale bar: 30 nm. Micrographs are low-pass filtered to 10 Å. (**B**) Representative 2D classes of a mixture of holo-TFIIH-XPC IEC, holo-TCD, and holo-TCDA complexes after blob picking.

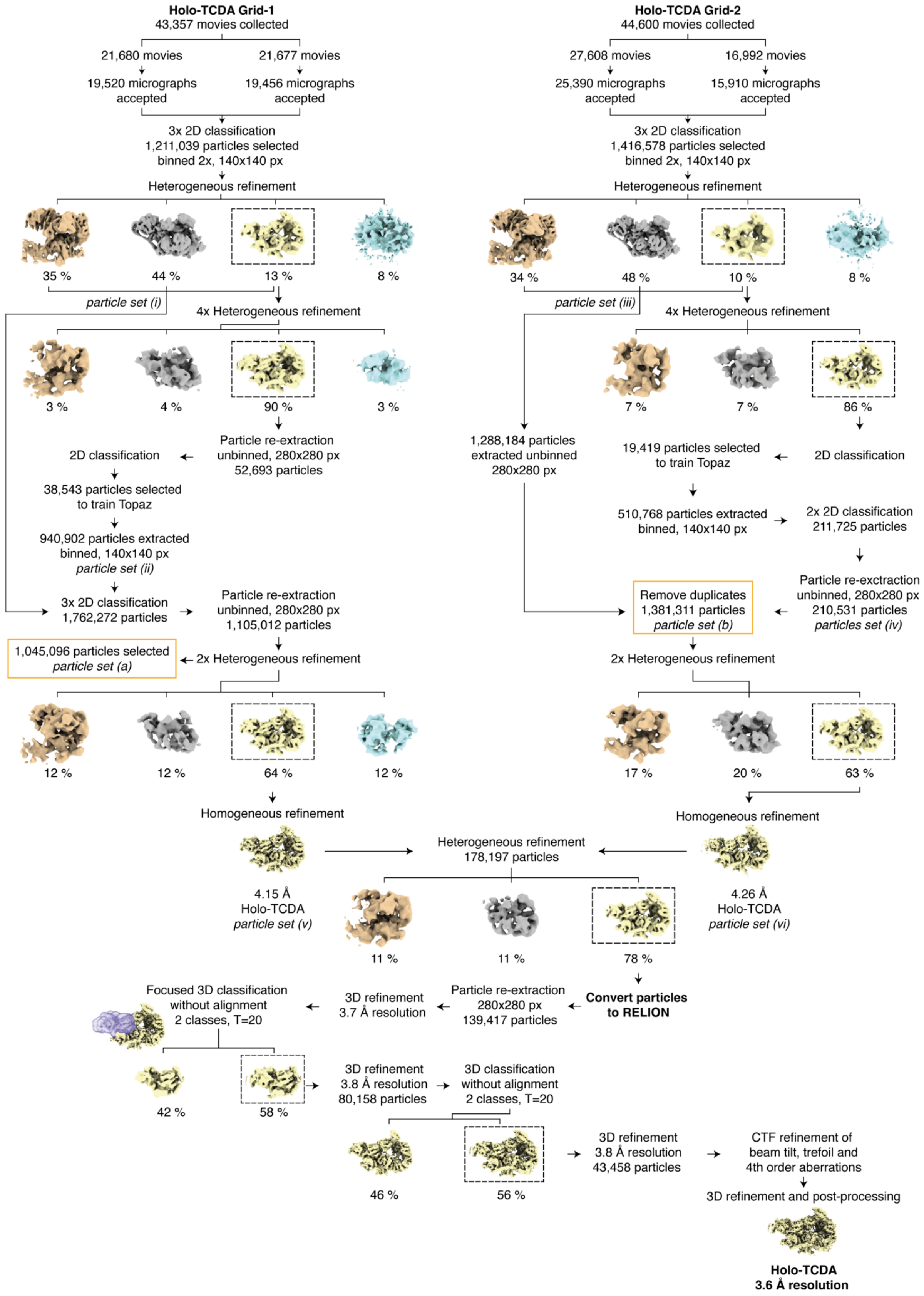

Figure S6. Cryo-EM data processing pipeline for the holo-TCDA complex final reconstruction from holo-TFIIH-XPCc-DNA-XPA grids. Cryo-EM processing pipeline used to obtain the final reconstruction of the holo-TCDA complex. Maps discussed in the main

text are labelled in bold. Particle distributions from classification are shown as percentages of total output particles. Classes which are taken forward for further processing are highlighted with dashed-line boxes. Particle sets used to obtain the final reconstructions of the holo-TFIIH-XPC IEC and the holo-TCD complex are highlighted in gold boxes. Masks used for focused 3D classification are shown as semi-transparent surfaces.

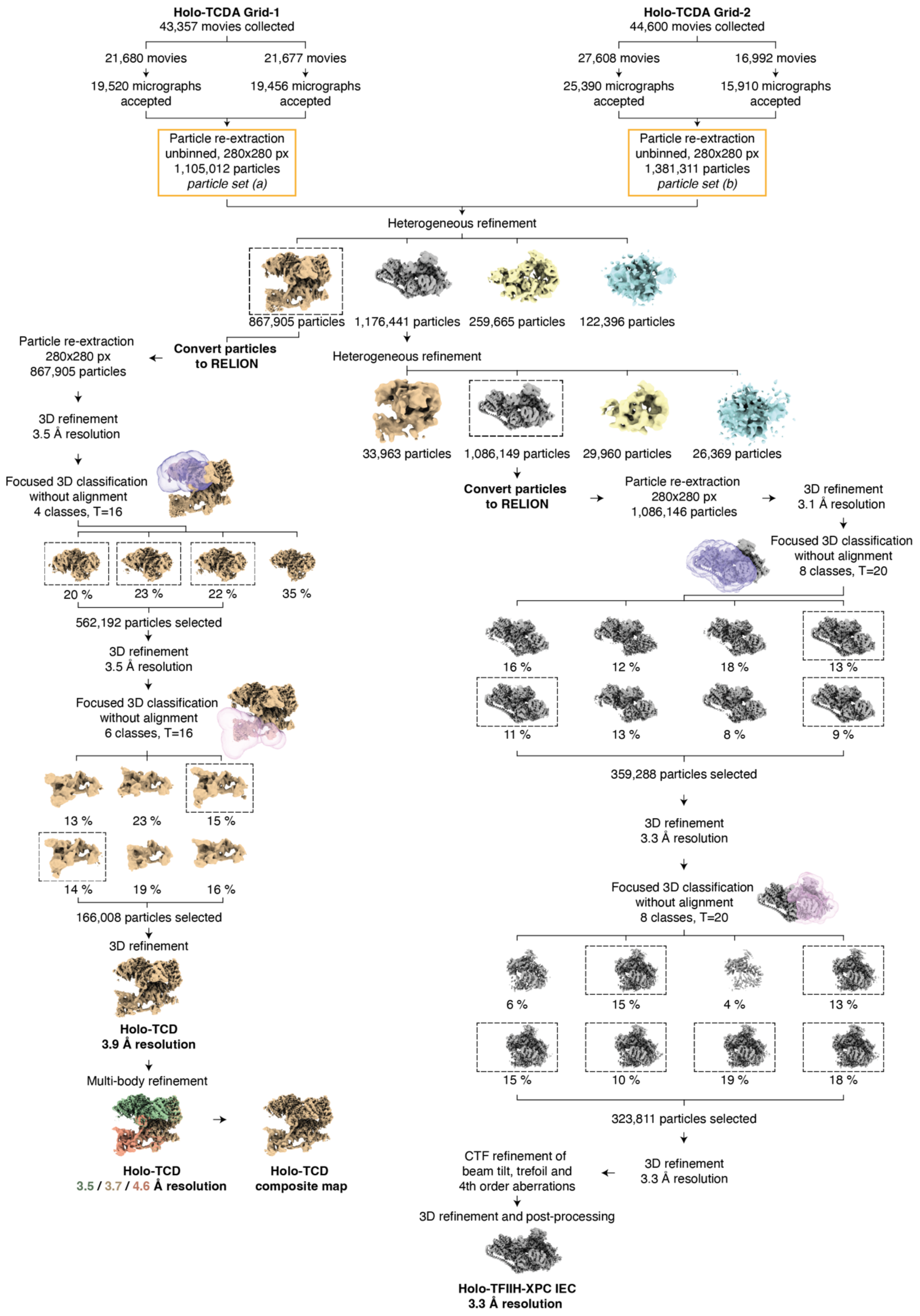

Figure S7. Cryo-EM data processing for the holo-TFIIH-XPC IEC and the holo-TCD complex final reconstructions from holo-TFIIH-XPCc-DNA-XPA grids. Cryo-EM processing pipeline used to obtain the final reconstruction of the holo-TFIIH-XPC IEC and

holo-TCD complex. Maps discussed in the main text are labelled in bold. Particle distributions from classification are shown as percentages of total output particles. Classes which are taken forward for further processing are highlighted with dashed-line boxes. The processing tree starts with particle sets obtained from processing of the holo-TCDA complex, which are highlighted in gold boxes here and in fig. S6. Masks used for focused 3D classification are shown as semi-transparent surfaces.

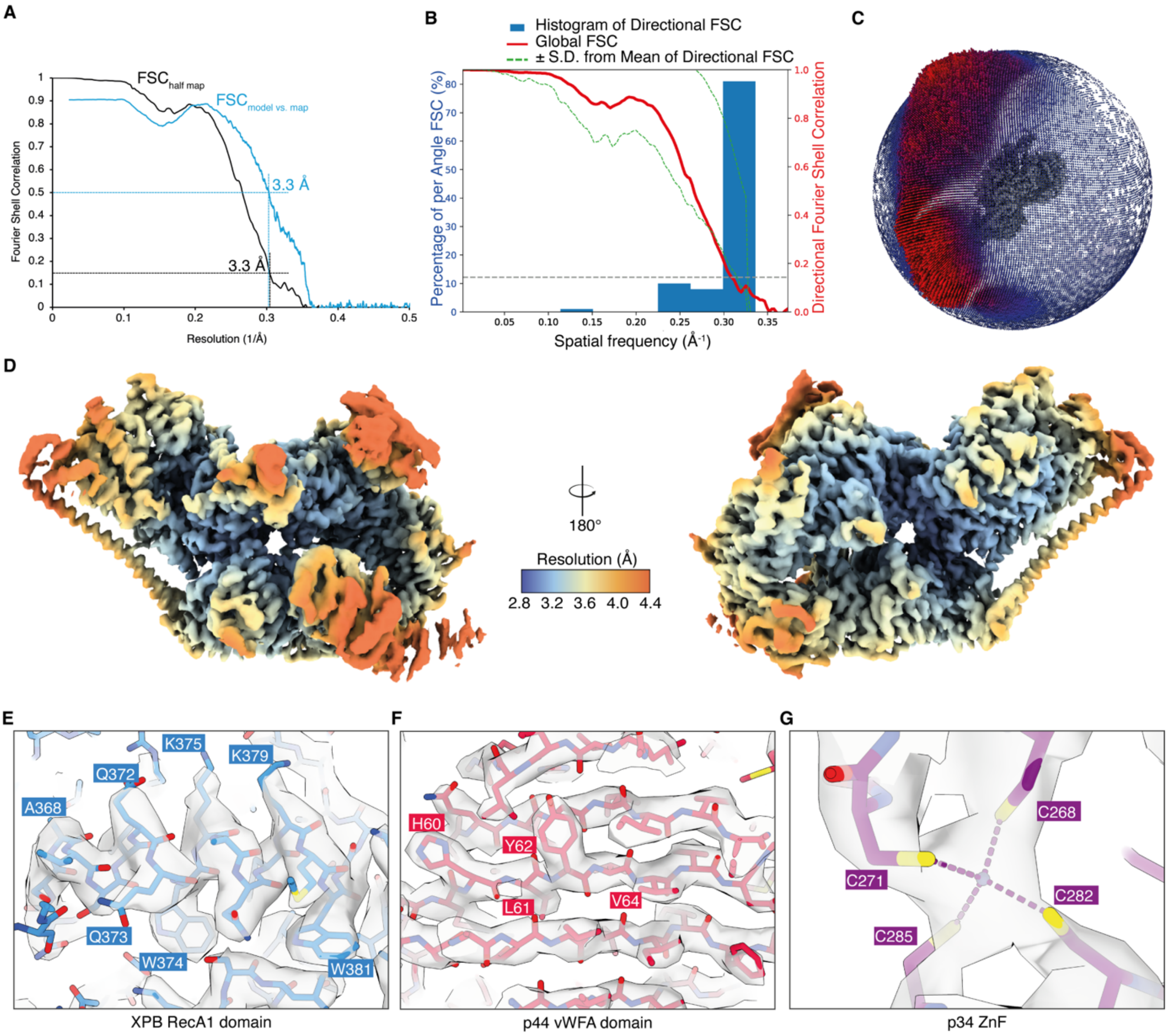

Figure S8. Overall and local resolutions of the holo-TFIIH-XPC IEC reconstruction. (**A**) Gold standard (black) and model vs. map (blue) FSC plots evaluated at the appropriate thresholds (*71*). (**B**) Directional FSC plot produced by the 3D FSC validation server (*80*). (**C**) Orientation distribution plot from RELION 3D auto-refinement. (**D**) Results from local resolution estimation computed in RELION. (**E-G**) Representative cryo-EM density of the XPB RecA1 domain, p44 von Willebrand factor type A (vWFA) domain, and p34 zinc finger (ZnF) domain.

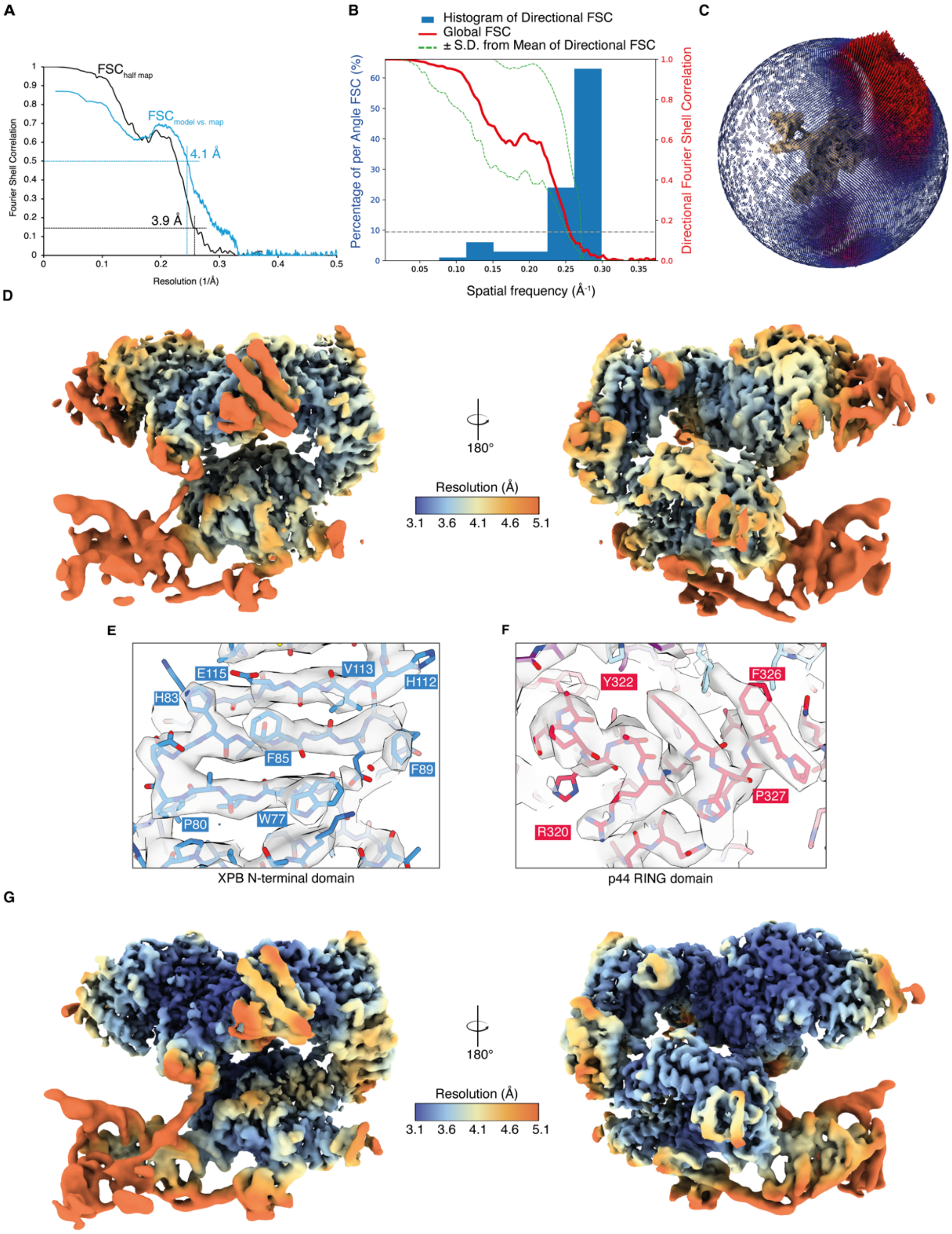

Figure S9. Overall and local resolutions of the holo-TCD reconstruction and comparison to the local resolution of the holo-TCD multibody reconstruction. (**A**) Gold standard (black) and model vs. map (blue) FSC plots evaluated at the appropriate thresholds (*71*). (**B**) Directional FSC plots produced by the 3D FSC validation server (*80*). (**C**) Orientation distribution plots from RELION 3D auto-refinement. (**D**) Results from local resolution estimation computed in RELION, mapped onto the local resolution-filtered holo-TCD consensus reconstruction. (**E**-**F**) Representative cryo-EM density of the XPB N-terminal and p44 RING domains in the holo-TCD consensus map. (**G**) Local resolution estimation computed using a composite map assembled from the three bodies of the the holo-TCD multi-body

refinement, mapped onto the local resolution-filtered composite map. The resolution scale is identical to panel D to facilitate comparison.

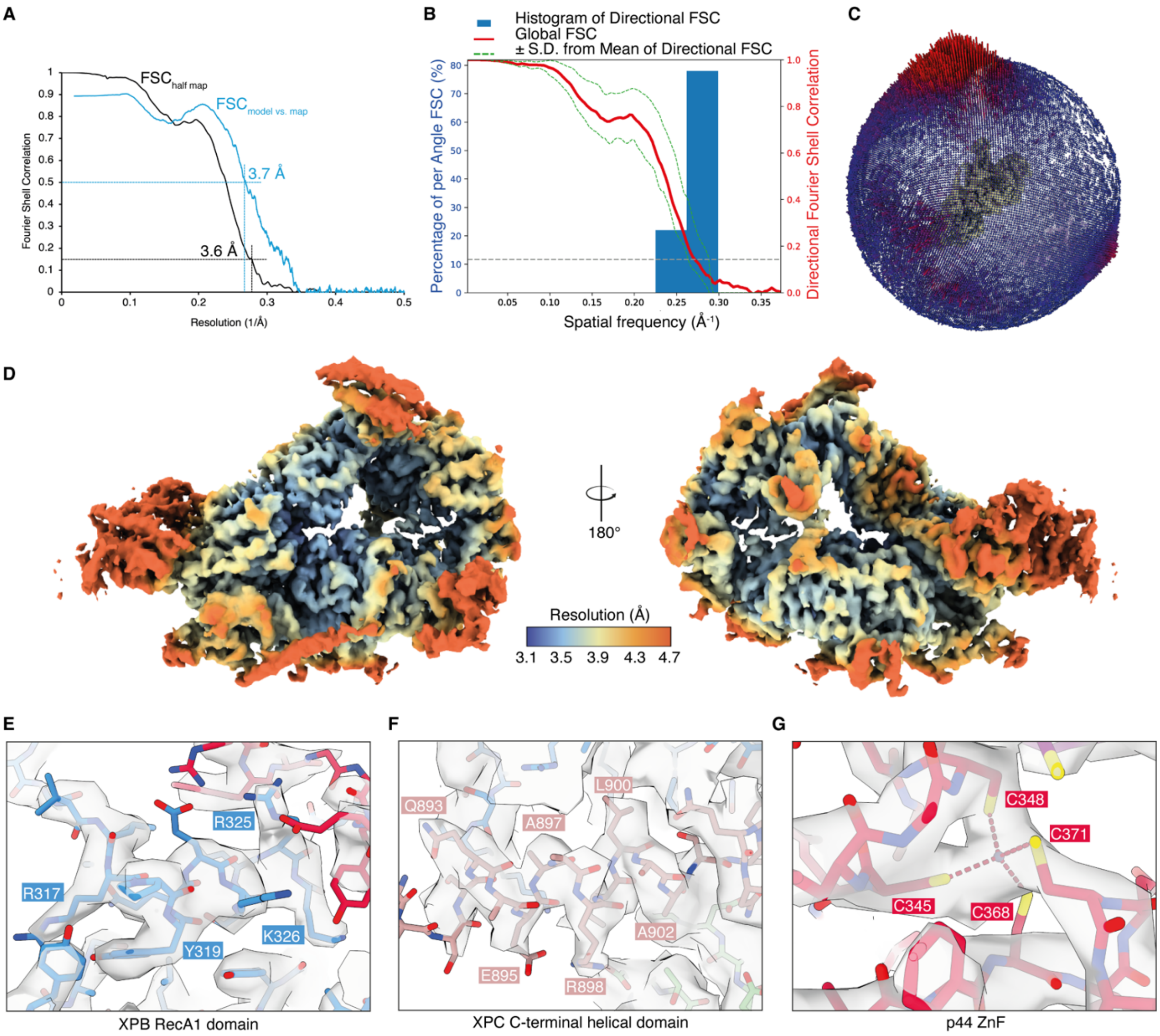

Figure S10. Overall and local resolutions of the holo-TCDA reconstruction. (**A**) Gold standard (black) and model vs. map (blue) FSC plot evaluated at the appropriate thresholds (*71*). (**B**) Directional FSC plots produced by the 3D FSC validation server (*80*). (**C**) Orientation distribution plot from RELION 3D auto-refinement. (**D**) Results from local resolution estimation computed in RELION. (**E**-**G**) Representative cryo-EM density of the XPB RecA1 domain, XPC C-terminal helical domain, and p44 zinc finger (ZnF) domain.

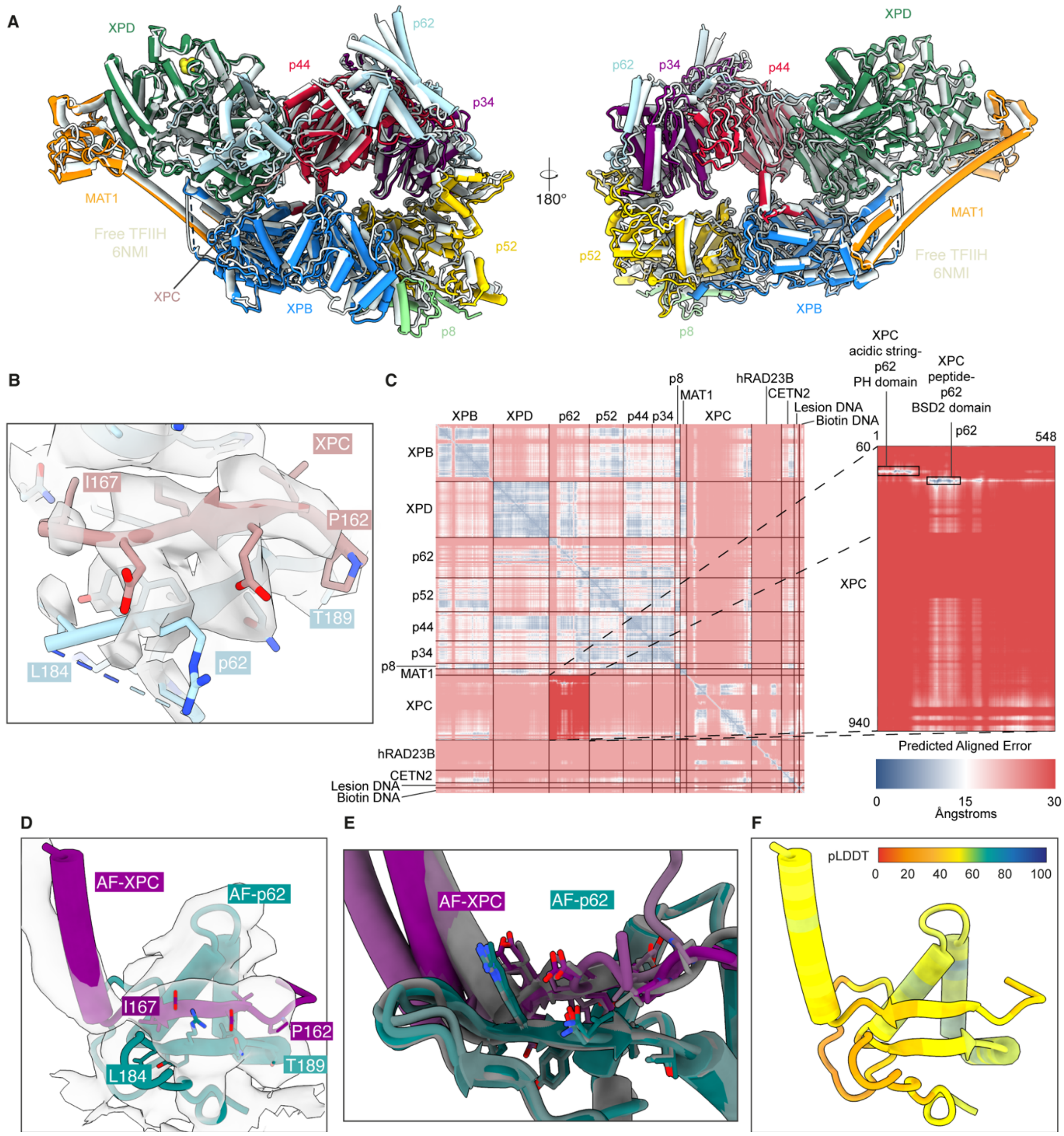

Figure S11. Structural comparison between hTFIIH-XPC IEC and free TFIIH and assignment of XPC segment to unassigned density. (**A**) Superposition of holo-TFIIH-XPC IEC and free holo-TFIIH (PDB 6NMI (*28*)) in cartoon representation. Holo-TFIIH-XPC IEC is coloured by subunit with metal ions and the FeS cluster depicted as spheres; free holo-TFIIH is coloured light grey. (**B**) Cryo-EM density and refined model for the interaction formed by XPC residues 162-167 and the p62 BSD2 domain. (**C**) Predicted aligned error (PAE) plot of for the Alphafold 3 prediction of the structure of the holo-TCD complex. The regions of the PAE plot corresponding to the XPC peptide-p62 BSD2 interaction and the XPC acidic string-p62 PH domain interactions are highlighted in black boxes in the inset and labelled. (**D**) Regions of XPC and p62 in the top-ranked AlphaFold 3 prediction corresponding to the XPC peptide-p62 BSD2 domain interaction (as labelled in panel C), fit into holo-TCD multi-body

map low-pass filtered to 6 Å. The p62 BSD2 domain and interacting region of XPC show excellent fit to the density, and and neighbouring predicted α-helix of XPC is resolved as well, albeit at a slightly different angle. (**E**) Superposition of XPC peptide-p62 BSD2 interaction of all five AlphaFold 3-predicted models with the top ranked model shown in teal and purple, and the other four models shown in hues with decreasing saturation. An anti-parallel β-sheet interaction between XPC residues 162-167 and the p62 BSD2 domain is consistently predicted. (**F**) XPC peptide-p62 BSD2 interaction prediction shown in panel D coloured by predicted local distance difference test (pLDDT) scores.

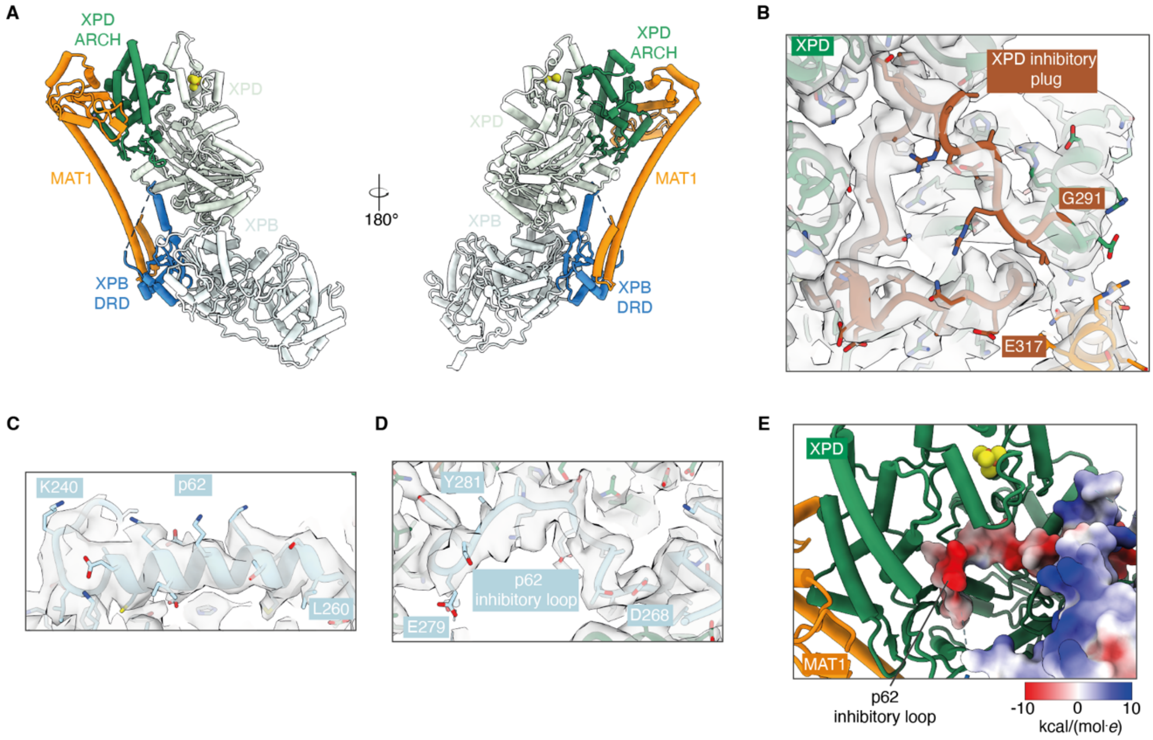

Figure S12. Structural details of the holo-TFIIH-XPC IEC. (**A**) Cartoon representation of the atomic model of XPB, XPD, and MAT1 in the holo-TFIIH-XPC IEC, highlighting the interaction between MAT1 and the XPD ARCH domain and the XPB DRD domain. (**B**) Cryo-EM density for XPD auto-inhibitory plug emanating from the XPD ARCH domain. (**C**) Cryo-EM density for the newly assigned residues of the p62 BSD2-inhibitory loop-linking helix (top) and (**D**) inhibitory loop (bottom). (**E**) Electrostatic surface representation of the p62 inhibitory loop interacting with XPD.

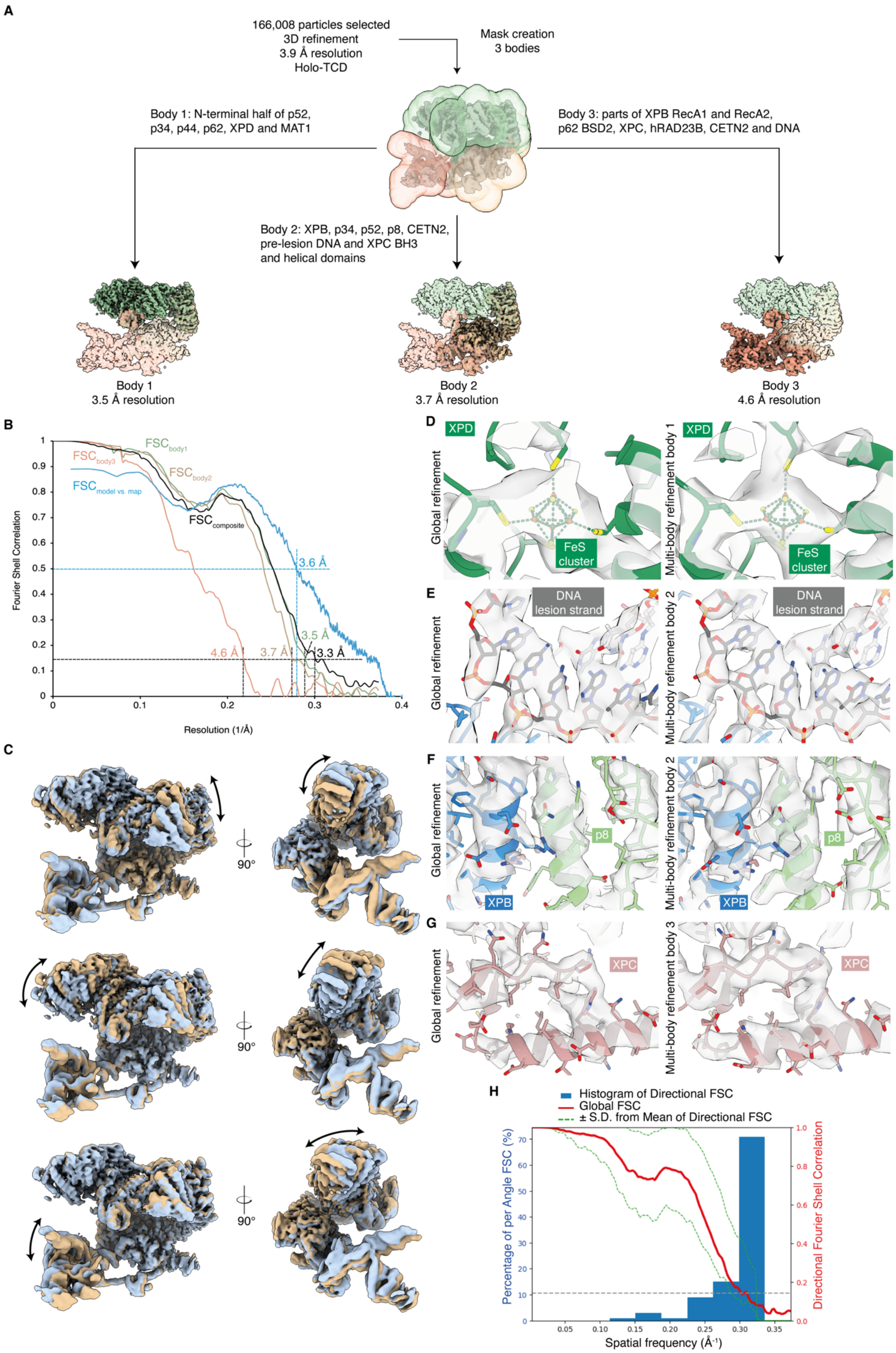

A
166,008 particles selected
3D refinement
3.9 Å resolution
Holo-TCD
Mask creation
3 bodies
Body 1: N-terminal half of p52, p34, p44, p62, XPD and MAT1
Body 2: XPB, p34, p52, p8, CETN2, pre-lesion DNA and XPC BH3 and helical domains
Body 3: parts of XPB RecA1 and RecA2, p62 BSD2, XPC, hRAD23B, CETN2 and DNA
Body 1
3.5 Å resolution
Body 2
3.7 Å resolution
Body 3
4.6 Å resolution
B
Fourier Shell Correlation
Resolution (1/Å)
FSCbody1
FSCbody2
FSCbody3
FSCmodel vs. map
FSCcomposite
3.6 Å
3.5 Å
3.3 Å
3.7 Å
4.6 Å
C
90°
90°
90°
D
Global refinement
Multi-body refinement body 1
XPD
FeS cluster
E
Global refinement
Multi-body refinement body 2
DNA lesion strand
F
Global refinement
Multi-body refinement body 2
XPB
p8
G
Global refinement
Multi-body refinement body 3
XPC
H
Histogram of Directional FSC
Global FSC
± S.D. from Mean of Directional FSC
Percentage of per Angle FSC (%)
Directional Fourier Shell Correlation
Spatial frequency (Å⁻¹)

Figure S13. Multi-body refinement of the holo-TCD complex. (**A**) Multi-body refinement strategy for reconstruction of the holo-TCD complex to high resolution. Masks used for body definition are shown as semi-transparency surfaces. (**B**) FSC plots for each multi-body-refined body along with the half-map and model vs. map FSCs for a composite map evaluated at the appropriate thresholds (*71*). (**C**) Principal component analysis from multi-body refinement in RELION showing the relative motions of each body. Direction of motion shown by arrows. (**D**-**G**) Comparison of map quality between global refinement and multi-body refinement of representative regions of each body. (**H**) Directional FSC plot of a composite map of the three bodies, produced in ChimeraX (*79*) and evaluated by the 3D FSC validation server (*80*).

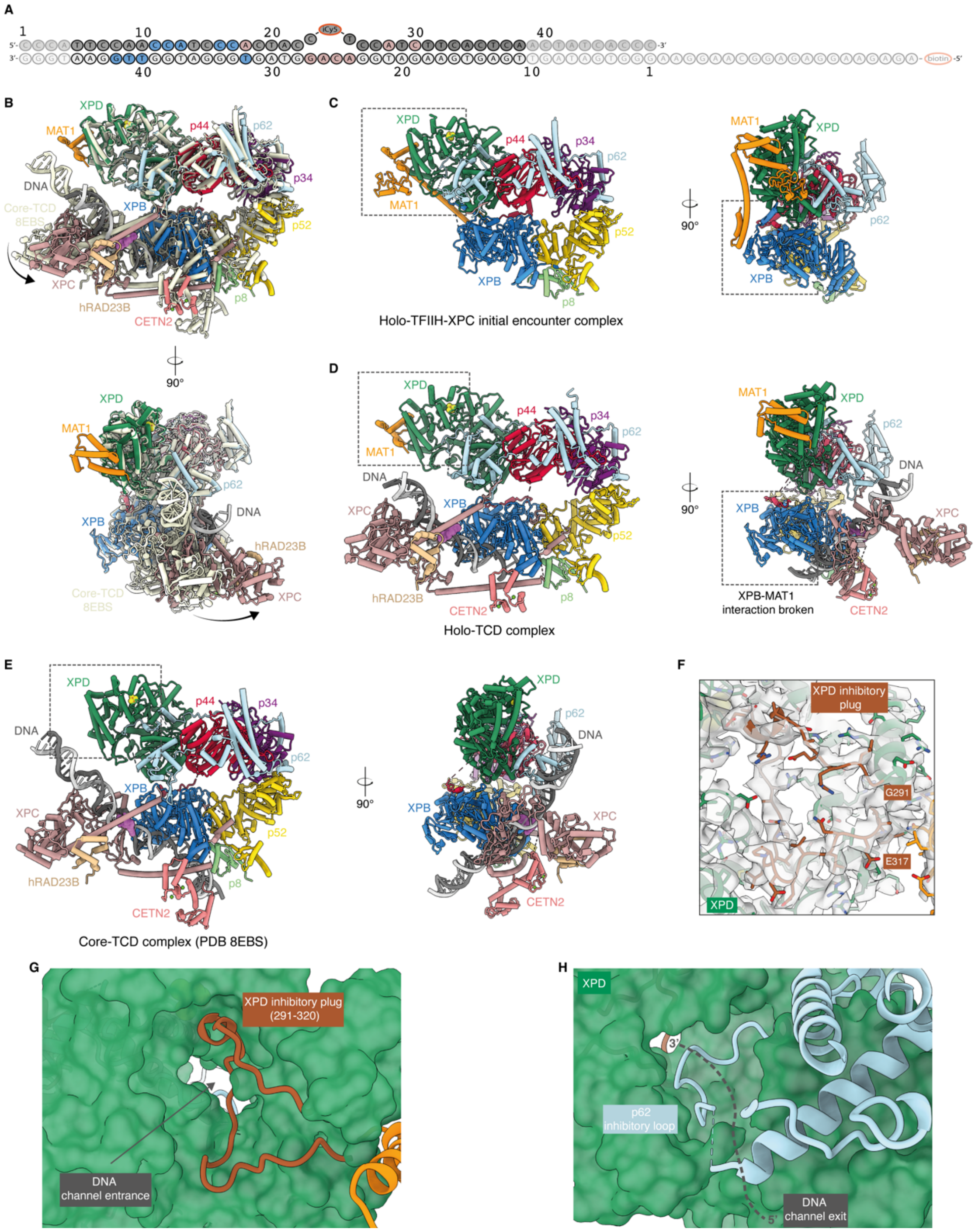

Figure S14. Structural details of the holo-TCD complex. (**A**) Schematic of biotinylated damaged DNA used to assemble and tether NER complexes to cryo-EM grids. Residues built in the holo-TCD model are shown as opaque. Nucleotides bound by protein in the multi-body composite model are highlighted in blue (XPB) or pink (XPC). (**B**) Superposition of the holo-TCD and core-TCD (*47*) complexes comparing the relative positions of the XPC complex and DNA, with shift shown by arrow. (**C-E**) Cartoon representations of the atomic models of the holo-TFIIH-XPC initial encounter complex (C), holo-TCD complex (D), and core-TCD complex (E, PDB 8EBS (*47*)), coloured by subunit and with ligands represented as spheres. Sites of MAT1-XPD and MAT1-XPB interaction are highlighted by dashed boxes for

comparison. (**F**) Cryo-EM density for the XPD auto-inhibitory plug in the holo-TCD complex. (**G**-**H**) Views from the entry (G) and exit (H) sites of the XPD DNA channel, with inhibitory regions shown as cartoons and XPD ARCH, FeS, and RecA2 domains shown as a surface.

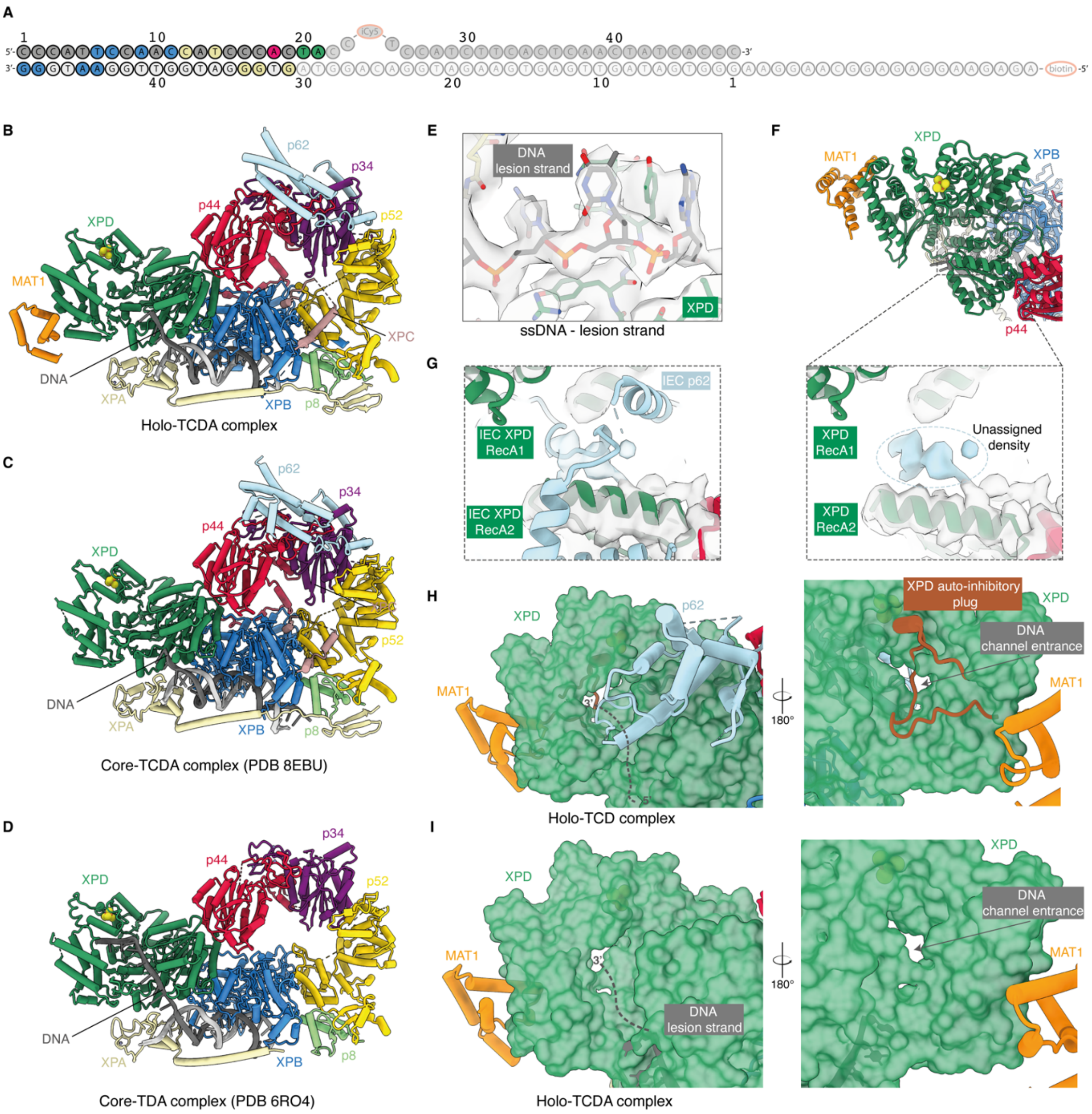

Figure S15. Structural details of the holo-TCDA complex. **(A)** Schematic of biotinylated damaged DNA used to assemble and tether NER complexes to cryo-EM grids. Residues built in the holo-TCDA model are shown as opaque. Nucleotides bound by protein are highlighted in yellow (XPA) blue (XPB), green (XPD), or fuchsia (XPA and XPD simultaneously). (**B**) Cartoon representation of the holo-TCDA, core-TCDA (C, PDB 8EBU (*47*)), and core-TDA (D, PDB 6RO4 (*25*)) complex, coloured by subunit and with ligands represented as spheres. (**E**) Cryo-EM density of single-stranded lesion strand DNA in contact with XPD. (**F**) Cartoon representation of the holo-TCDA model focusing on XPD, coloured by subunit and with ligands represented as spheres. Inset shows unassigned density found on the XPD RecA2 domain coloured in light blue. The cryo-EM map was low-pass filtered to 4 Å for visualisation. (**G**) Cartoon representation of the holo-TFIIH-XPC IEC structure, positioned by superposition on the holo-TCDA model, showing that p62 in the holo-TFIIH-XPC-IEC occupies the same position as the unassigned density in the holo-TCDA but does not fit the density in this conformation. (**H-I**) Comparison of the DNA exit (left) and entry (right) sites of the XPD channel of the holo-TCD (top) and holo-TCDA (bottom) complexes.

Table S1. Cryo-EM data collection, refinement, and validation statistics.

Dataset	holo-TFIIH-XPC IEC	holo-TCD consensus	holo-TCD composite	holo-TCDA
Microscope	Titan Krios G3i			
Stage type	Autoloader			
Voltage (kV)	300			
Detector	Gatan K3			
Energy Filter	Bio Quantum			
Acquisition mode	Super-resolution (grid-2) / counting (grid-1) with 2x hardware binning			
Pixel size (non-superresolution) (Å)	0.67 (grid-2) / 1.34 (grid-1)			
Defocus range (µm)	0.6-2.5			
Electron exposure (e^{-}/A^2)	60			
**Reconstruction**	**EMD-56544**	**EMD-56566**	**EMD-56551**	**EMD-56573**
Software	RELION 5.0.0 CryoSPARC v4.6.2	RELION 5.0.0 CryoSPARC v4.6.2	RELION 5.0.0 CryoSPARC v4.6.2	RELION 5.0.0 CryoSPARC v4.6.2
Particles after 2D classification	2,426,407	2,426,407	2,426,407	2,486,323
Particles final reconstruction	323,811	166,008	166,008	43,458
Extraction box size (pixels)	280 x 280 x 280	280 x 280 x 280	280 x 280 x 280	280 x 280 x 280
Final pixel size (Å)	1.34	1.34	1.34	1.34
Accuracy rotations (°)	0.779	1.016	-	1.134
Accuracy translations (Å)	0.402	0.579	-	0.536
Map resolution (Å)	3.3	3.9	3.3	3.6
Map resolution range (Å)	2.9-5.0	3.0-8.0	2.7-8.0	3.1-5.5
Sphericity (3D FSC)	0.868	0.798	0.818	0.891
Map sharpening B-factor ($Å^2$)	-10	-40	-	-20
**Coordinate refinement**				
Software and algorithm	PHENIX (real space refine)	PHENIX (real space refine)	PHENIX (real space refine)	PHENIX (real space refine)
Resolution cutoff (Å)	3.3	3.9	3.3	3.6
$FSC_{model-vs-map}$=0.5 (Å)	3.3	4.1	3.6	3.7
**Model**	**PDB-28JM**	**PDB-28JV**	**PDB-28JS**	**PDB-28KE**
Number of residues				
Protein	3147	3648	3648	2893
Nucleic Acids	-	68	68	40
B-factors overall	105.25	84.85	140.28	106.56
Protein	105.23	82.95	139.91	106.57
Nucleic Acids	-	122.51	146.96	104.37
Ligands	139.54	146.84	168.69	217.22
R.M.S. deviations				
Bond lengths (Å)	0.002	0.004	0.003	0.003
Bond angles (°)	0.542	0.723	0.606	0.570
**Validation**				
Molprobity score	1.79	2.16	1.76	2.00
Molprobity clashscore	7.83	12.57	8.46	8.96
Rotamer outliers (%)	1.53	1.98	1.15	2.26
C_{β} deviations (%)	-	-	-	-
Ramachandran plot				
Favored (%)	96.56	95.36	96.25	96.22
Allowed (%)	3.41	4.64	3.75	3.78
Outliers (%)	0.03	0.00	0.00	0.00
Rama-Z scores				
Whole	0.42	-0.58	0.15	0.56
Helix	1.92	0.55	1.15	2.06
Sheet	-0.94	-0.93	-0.30	-0.31
Loop	-1.53	-1.30	-0.95	-1.67

Table S2: Validation statistics for the three multi-body maps.

**Reconstruction**	**hTCD body 1 EMD-56553**	**hTCD body 2 EMD-56568**	**hTCD body 3 EMD-56572**
Software	RELION 5.0.0<br>CryoSPARC v4.6.2	RELION 5.0.0<br>CryoSPARC v4.6.2	RELION 5.0.0<br>CryoSPARC v4.6.2
Particles after 2D classification	2,426,407	2,426,407	2,426,407
Particles final reconstruction	166,008	166,008	166,008
Extraction box size (pixels)	280 x 280 x 280	280 x 280 x 280	280 x 280 x 280
Final pixel size (Å)	1.34	1.34	1.34
Accuracy rotations (°)	1.384	1.301	2.681
Accuracy translations (Å)	0.541	0.636	1.311
Map resolution (Å)	3.5	3.7	4.6
Map sharpening B-factor ($Å^2$)	-40	-40	-180

Table S3. Summary of modelled regions of the holo-TFIIH-XPC-IEC, holo-TCD and holo-TCDA complexes.

Complex	Protein subunits	Length (residues)	Modelled			Chain ID
			Holo-TFIIH-XPC IEC	Holo-hTCD	Holo-TCDA	
Holo-TFIIH	XPB	782	36-202, 248-719, 771-780	42-213, 265-719, 772-780	43-208, 267-646, 654-722, 771-780	A
	XPD	760	1-760	1-422, 428-760	1-289, 318-420, 429-730	B
	p62	548	115-146, 161-176, 184-285, 292-321, 346-442, 452-548	115-146, 161-176, 183-286, 294-321, 346-442, 452-548	397-548	C
	p52	462	9-458	9-295, 301-462	17-292, 304-462	D
	p44	395	14-43, 49-387	9-28, 48-387	8-43, 53-388	E
	p34	308	8-72, 95-292	6-72, 95-151, 156-292	6-73, 98-289	F
	p8	71	2-67	2-71	2-69	G
	MAT1	309	1-210	64-140	66-140	H
XPC complex	XPC	940	162-167	162-319, 530-866, 889-916, 930-940	889-916, 928-940	I
	hRAD23B	409	-	271-331	-	J
	CETN2	172	-	98-168	-	K
XPA	XPA	273	-	-	102-272	N
DNA	Damaged strand	1 to 48	-	5-23-iCy5-25-38	1-21	L
	Non-damaged strand	-20 to 49	-	11-45	31-49	M

Table S4. Key structural features of the holo-TFIIH-XPC IEC, holo-TCD and holo-TCDA complexes.

Holo-TFIIH-XPC IEC	Holo-TCD	Holo-TCDA
Not bound to DNA	XPB contacting dsDNA upstream of the lesion	Both XPB and XPD contacting DNA: XPB contacts dsDNA while XPD starts engaging with ssDNA
XPD auto-inhibited by auto-inhibitory plug and p62-blocking fragments	XPD auto-inhibited by auto-inhibitory plug and p62-blocking fragments	XPD de-repressed: auto-inhibitory plug and p62-blocking fragments displaced
MAT1 interacting with both XPB and XPD	MAT1 helical bundle visualised contacting XPD only	MAT1 helical bundle visualised contacting XPD only
XPB and XPD contacting each other	XPB and XPD contacts broken as holo-TFIIH adopts a wider conformation	XPB and XPD contacting each other through a different interface as holo-TFIIH adopts a sigma-shape conformation
XPC tethered to holo-TFIIH by a short segment interacting with p62 BSD2 domain	Trimeric XPC complex visualised (XPC, hRAD23b, CETN2) with XPC bound to the DNA	XPC tethered to holo-TFIIH through its C-terminal helical domain
XPA not visualised	XPA not visualised	XPA visualised bound to DNA and TFIIH

Table S5 (separate file)
List of synthetic DNA used in this study.

Data S1 (separate file)
AlphaFold 3 web server output for the prediction of the structure of the holo-TCD complex, including all data files for validation.
